# Smoking reduction trajectories and their association with smoking cessation: a secondary analysis of longitudinal clinical trial data

**DOI:** 10.1136/bmjph-2024-001605

**Published:** 2025-12-25

**Authors:** Anthony Barrows, Elias Klemperer, Hugh Garavan, Nicholas Allgaier, Nicola Lindson, Gemma Taylor

**Affiliations:** 1 Psychiatry, University of Vermont, Burlington, Vermont, USA; 2 University of Oxford, Oxford, UK; 3 Centre for Public Health, Population Health Sciences, Bristol Medical School, Medical Research Council Integrative Epidemiology Unit at the University of Bristol, University of Bristol, Bristol, UK; 4 Department of Psychology, University of Bath, Claverton Down, UK

**Keywords:** Public Health, Sociodemographic Factors, methods

## Abstract

**Introduction:**

Tobacco smoking remains the leading cause of preventable death worldwide. Smoking reduction can be recommended to people unmotivated to quit, but evidence on trajectories of reduction and associated outcomes is mixed.

**Methods:**

In a secondary analysis of five randomised, placebo-controlled trials of nicotine replacement therapy, we used latent class analysis and elastic net regression to determine latent smoking trajectories using cigarettes-per-day (CPD) across 26 weeks. Participants were adults who smoked daily without intention to quit in the next month. We used predictive modelling and receiver operator characteristic area under-the-curve (AUC) to assess smoking cessation after 1 year.

**Results:**

Participants (n=2066) smoked a mean 27.26±9.74 CPD at baseline. Three distinct smoking patterns emerged: Class 1 (n=186, 10%) achieved the greatest reduction in CPD (2-week mean 57% reduction) with subsequent reduction; Class 2 (n=803, 45%) saw a 2-week mean 50% reduction and remained at that level and Class 3 (n=794, 45%) reduced by a 2-week mean of 22% and returned to near-baseline CPD. Older, male participants with lower anxiety and lower nicotine dependence were more likely to be in Class 1. Abstinence rates at 1 year (~50 weeks after reduction) were 37.6% for Class 1, 4.2% for Class 2 and 2.3% for Class 3.

Using latent class assignment as a predictor improved prediction of smoking cessation at 1 year follow-up over prediction using baseline characteristics by 14.4% (AUC=0.776±0.010, p=0.002). Those who reduced their CPD minimally were nearly 90% less likely to achieve cessation than those who reduced by over 50% (ORs: Class 2=0.111±0.013, Class 3=0.070±0.005).

**Conclusions:**

Findings suggest adults who are unmotivated to quit at baseline but reduce their smoking by more than half are most likely to achieve smoking cessation. A lack of early reduction success could indicate that greater support is needed to help people to quit.

**WHAT IS ALREADY KNOWN ON THIS TOPIC**

- Currently, little is known about whether people asked to reduce their smoking do so differently from one another, and whether distinct characteristics and behaviours predict differences in smoking reduction.

**WHAT THIS STUDY ADDS**

- The present work identifies trajectories of smoking reduction in a sample of people unmotivated to quit, the magnitude of smoking reduction (ie, more than 50%) most associated with complete cessation and the participant characteristics associated with different smoking trajectories.

**HOW THIS STUDY MIGHT AFFECT RESEARCH, PRACTICE OR POLICY**

- Our findings demonstrate the importance of smoking reduction during the first 2 weeks of an intervention and provide information to help clinicians identify people who are most likely to achieve complete cessation and those in need of additional support.

## Introduction

 Smoking is the number one cause of premature and preventable illness worldwide.^1^ Smoking accounts for approximately 5.7% of total health expenditure worldwide and 6.5% in high-income countries like the UK and USA.^2^ Approximately 50% of people who smoke will die from tobacco-related illness unless they quit.^3^

Pre-cessation smoking reduction is sometimes recommended to people who have found it difficult to quit smoking abruptly in the past. Though magnitude and duration vary, broadly, smoking reduction is defined as a decrease in the number of cigarettes smoked per day. In the UK and in some parts of Europe, smoking reduction is promoted as a second-line route to quitting smoking or as a harm reduction approach for people who cannot, or may not be ready to, stop smoking completely.^4 5^ In addition, nicotine replacement therapy (NRT) is licensed for smoking reduction in Europe.^6^ In the USA, smoking reduction is common among those unable or unmotivated to quit abruptly, and NRT is deemed appropriate for use as a reduction aid.^7^ There is limited evidence that reduction itself improves health.^8^ However, there is clearer evidence that smoking reduction is as effective as abrupt quitting in achieving complete cessation,^9^ which has well-established health benefits.

Smoking reduction is common and those who smoke often report that they perceive reduction as an acceptable way to stop smoking.^10^,^14^ Those wishing to reduce their daily number of cigarettes could use a range of different approaches, including pharmacotherapy,^9^ structured behavioural strategies^15 16^ or simply reducing as much as possible without any specific guidance on how to do so.^17^,^21^ Regardless of approach, little is known regarding the trajectories by which people change their smoking when trying to reduce. Perhaps most importantly, research is needed to investigate the amount of smoking reduction achieved and whether greater magnitudes of reduction predict greater smoking cessation. Furthermore, it is unknown whether patterns of smoking reduction are influenced by individual characteristics or dependence severity. This information could be important to those developing and tailoring smoking reduction interventions, and it could aid clinicians who recommend and offer smoking reduction treatment.

To explore potential groups of people who reduce smoking differently from one another, we analyse data from five randomised placebo-controlled trials of NRT for smoking reduction. In particular, we (1) use latent class analysis (LCA) to identify trajectories in cigarettes-per-day (CPD) over time in people who are asked to reduce their smoking before quitting without being given specific instructions on how to do so, (2) explore predictors of these trajectories using elastic net regression and (3) determine how pre-cessation reduction predicts smoking cessation.

LCA^22^ is a person-centred mixture modelling technique which presumes that there are unobserved (or latent) subpopulations based on patterns in observed responses, and that respondents’ population membership can be determined analytically.^23^ Although several studies have used LCA to explore heterogeneity in smoking behaviour trajectories,^17^,^19^ none to date have leveraged contemporary machine learning (ML)-based methods to determine characteristics associated with latent class membership. The present study uses regularised linear models to minimise noise associated with highly correlated predictors while emphasising effects from relevant predictors of class membership and smoking cessation. These methods focus primarily on overall predictive performance rather than statistical significance in an effort to capture nuanced use patterns as compared with simply more-versus-less reductions in CPD.^24^

## Methods

This study was preregistered on Open Science Framework,^25^ and all analytical code is available through GitHub.^26^

### Study design

This secondary analysis examined individual-level patient data from five randomised placebo-controlled trials of NRT for smoking reduction.^17^,^19^ The trials were carried out to a consistent protocol. Participants were provided with active NRT (inhaler or gum) in the intervention arms and a placebo form of the same NRT in the control arms. In both arms, participants received the same minimal level of behavioural support for smoking reduction (ie, they were simply encouraged to reduce their smoking as much as possible), with cessation as the end goal. Each trial’s primary outcome was a 50% reduction in CPD from baseline.

The trials were conducted and funded by McNeil AB who manufacture NRT. Study protocols were consistent across trials but registry numbers were unavailable for all but one study: 980-CHC-9021–0013.^17^ The current authors were not involved with the original trials, and the funder was not involved with the planning, analysis, interpretation or funding of this study.

### Study details

**Figure 1:**
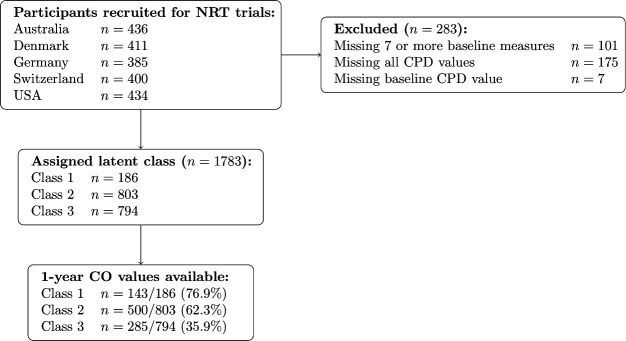
Participant record availability flowchart. Participants with baseline CPD values and at least one follow-up value were included in the analysis of smoking trajectories. For smoking cessation analysis, participants missing CO values were assumed to have continued smoking. CO, carbon monoxide; CPD, cigarettes-per-day; NRT, nicotine replacement therapy.

The trials took place between 1997 and 2003 and were conducted in university and medical centres in Denmark,^21^ Switzerland,^19^ Australia,^17^ the USA^20^ and Germany.^18^ There were 2066 participants enrolled across all five trials. At baseline, all participants were ≥18 years old, reported smoking ≥15 CPD, were selected because they wanted to reduce but had no plans to stop smoking in the next month (ie, responded ‘No’ to the question ‘Do you intend to quit smoking completely in the next month?’) Additionally, all participants had smoked regularly for ≥3 years and had made at least one failed quit attempt in the 2 years prior to baseline. Participants were excluded if they were pregnant, breastfeeding, under psychiatric care, deemed to be unfit by a general practitioner or currently enrolled in a smoking cessation programme (see figure 1).

### Patient and public involvement

Patients who wanted to reduce their smoking were recruited for participation in the included trials, and all participants were given behavioural support to do so.

### Data collection

At baseline, trial investigators gathered data on participants’ demographic details, age they started smoking, nicotine dependence, intention to reduce, motivation to quit, smoking history (number of previous quit attempts, longest period without smoking, time since last quit attempt), self-rated effects from smoking and self-reported physical and emotional health. Motivation to quit smoking (ie, ‘How much would you say you want to stop smoking?’) was assessed differently from the trial exclusion criterion of intention to quit smoking within the next month (ie, ‘Do you intend to quit smoking completely in the next month?’). To preserve anonymity, some demographic data were unavailable for this secondary analysis.

Participants also provided a breath carbon monoxide (CO) sample and answered the following questions at baseline and at 2, 10, 18, 26 and 52 weeks from baseline: ‘how many cigarettes do you smoke/day on average?’, ‘how many cigarettes do you smoke/week on average’ and ‘how many cigarettes do you smoke/month on average?’

Latent trajectories were determined using per cent change from baseline in average CPD at weeks 2, 10, 18 and 26. When CPD was unavailable, participants’ self-reported values for cigarettes-per-week were divided by 7. If a participant reported they had stopped smoking, CPD was set to 0. Abstinence at 52 weeks was determined using CO values <6 parts per million (ppm), consistent with recent guidance.^27^ See online supplemental file 1 for cessation prediction with the threshold used at the time of the trial.

The baseline variables used to predict latent class were age at trial intake, age started smoking, longest period without smoking, number of previous quit attempts (Fagerström Test of Nicotine Dependence; FTND),^28^ motivation to quit, length of time since last quit attempt, experiences of anxiety and depression, the Short Form Health Survey-36 (SF-36/RAND-36)^29^,^31^ subscales, CO ppm, relief from smoking questionnaire (RSQ), study site and trial treatment group (22 variables). Coding information for baseline variables is included in online supplemental materials.

With regard to missing data, two baseline SF-36 subscales (General Health and Physical Functioning) were discarded due to missing greater than 25% of observations. Additionally, 101 participants were missing ≥7 baseline variables and thus were excluded from analyses. Seven participants were missing baseline CPD values, and an additional 175 participants were missing all CPD values between baseline and week 26 and were thus excluded from analyses. The 1783 remaining subjects had at least one post-baseline average CPD value. This cautious approach was selected to help overcome bias introduced through non-random missingness.^32^

The remaining missing values for baseline variables (ie, RSQ pepping-up and calming effects of smoking, and overall last cigarette experience: 6.6%, 1.4% and 0.1% missing, respectively; motivation to quit: 2.6% missing; SF-36 subscales: <1% missing) were stratified by age (grouped into four equally sized partitions), sex and study site, and then imputed using the mean (for continuous variables) and mode (for categorical variables) within those groups.

### Statistical methods

**Figure 2:**
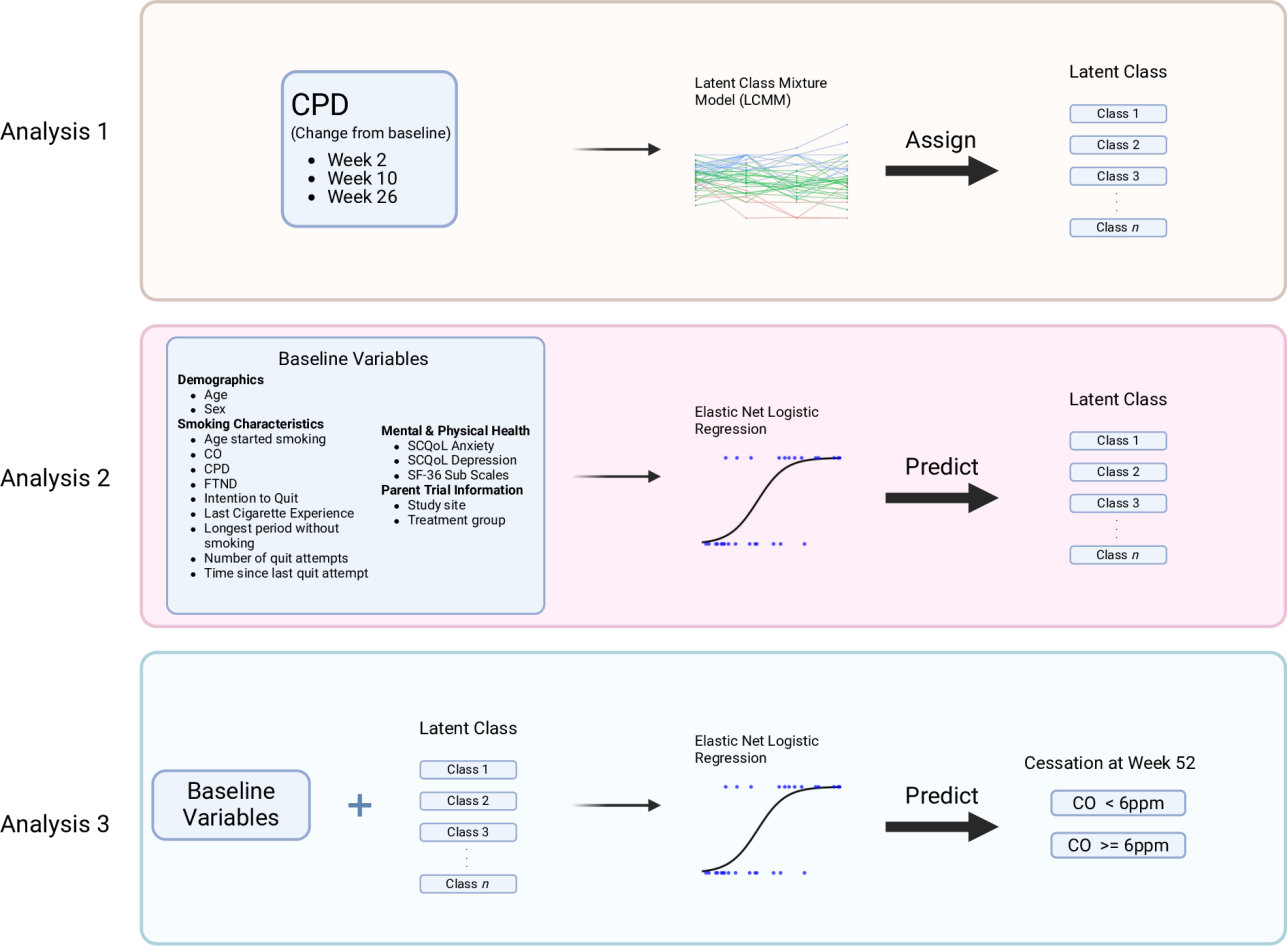
Overview of methods. Analysis 1: trends in CPD are determined using Latent Class Mixture Models. Analysis 2: class membership is predicted through elastic net regression using baseline and demographic variables. Analysis 3: post-trial smoking cessation is predicted through elastic net regression using the same baseline and demographic variables as in analysis 2, plus latent class membership from the results of analysis 1. CO, carbon monoxide; CPD, cigarettes-per-day; FTND, Fagerström Test for Nicotine Dependence; SCQoL, Smoking Cessation Quality of Life Questionnaire; SF-36, Short Form Health Survey-36.

The present work (1) uses latent class mixture models^33^,^35^ to uncover latent trends in cigarette usage in the parent clinical trials independent of treatment group assignment; (2) employs elastic net logistic regression to determine which baseline and demographic characteristics are most associated with these trajectories and (3) fits additional elastic net regression models to determine whether knowledge of a participant’s smoking trajectory improves prediction of cessation after the trial. See figure 2 for details.

#### Analysis 1: trajectories in CPD over time

The goal of this analysis was to identify possible trajectories in CPD use, and participants could be grouped according to those trends. A latent class mixture model^33^,^35^ was used to determine longitudinal trends in CPD from baseline assessed at trial weeks 2, 10, 18 and 26. The primary dependent variable was per cent change in average CPD (eg, a participant smoking 90% of their baseline level at week 2 received a value of −10% for week 2). Trajectories were modelled using CPD rather than breath CO because of the measure’s intuitive and tractable interpretation for clinical use without the need for specialised equipment.

Models ranging from 1 to 6 classes were estimated, and models for classes >1 were initialised using parameters from the 1-class model. Grid search methods were performed for subsequent models, seeking to minimise Bayesian information criterion (BIC) for that model at each step. BIC has been shown to favour parsimonious models and allows for direct comparisons between latent class models.^23^ However, the optimal model is one that considers objective fit statistics, posterior classification quality (ie, separability of cigarette use trajectories) and interpretability of the classes.^36^ The model that balanced fit, parsimony and interpretability was selected as optimal (see online supplemental sFigure 9 for alternative models).

Posterior classification was used to assign subjects to each latent class using maximum likelihood estimation given the information (ie, per cent change in CPD at each week) collected in the longitudinal model. See online supplemental materials for additional information on latent class model fitting.

#### Analysis 2: predicting longitudinal trajectories in CPD using baseline variables

The goal of this analysis was to identify predictors of each trajectory group (latent class) in order to investigate the association between participant characteristics and smoking trajectories. Elastic net logistic regression^37 38^ was used to build predictive models for each latent class using all baseline characteristics as features (ie, independent variables) and class membership as the target (ie, dependent variable). Class membership was treated as a binary outcome, with membership to each class predicted using a separate regression model.

Initially, the data were split into 80% training and 20% testing sets. The training data were then used to tune parameters for elastic net logistic regression models. Model selection and evaluation was performed using a nested cross-validation framework. Training was conducted using fivefold cross-validation, with 80% of the training set used for model training and 20% for evaluation. Model hyperparameter selection was conducted using a further divide of this training set using fivefold cross-validation within each outer fold. Prediction accuracy was measured using receiver operator characteristic area-under-the-curve (ROC AUC). Parameters from the model whose ROC AUC results were within 1SE of the optimal cross-validated results were selected.^39^ For each model, ROC AUC represents the accuracy of prediction of the internal validation set. Hyperparameters from the most successful of these models were used to fit a model to the initial training set to obtain coefficients. To confirm model generalisability, these hyperparameters were also used to fit a final model to the initial testing set. Null ROCs were computed for each predicted class, and overall statistical significance was assessed using ROC AUC’s equivalence with the Mann-Whitney U statistic.^40^ This procedure was repeated using each latent class as the target. See supplemental materials for sensitivity analyses using alternative data splits (online supplemental sFigures 10 and 11), and additional details on hyperparameter selection.

#### Analysis 3: which trajectories in CPD predict smoking cessation?

Our third goal was to determine which, if any, smoking trajectory groups were most associated with smoking cessation. Elastic net logistic regression was used to predict smoking abstinence at 52-week follow-up using (1) all baseline characteristics from Analysis 2, (2) latent class assignment (from Analysis 1) in addition to those predictors and (3) baseline predictors with only subjects in each latent class, respectively. The dependent variable was biochemically verified smoking cessation. Participants were counted as having quit smoking if they reported doing so and registered a CO value of <6 ppm.^27^ All other participants were counted as continuing to smoke. Each regression model was fit and evaluated using nested cross-validation procedures described in Analysis 2.

### Quantitative variables

Ordinal predictors (ie, longest period without smoking, number of times tried to quit smoking, motivation to quit, length of time since last quit attempt, experience of anxiety in the last 24 hours, experience of depression in the last 24 hours and relief from smoking) were treated as numeric variables. These plus additional numeric predictors (ie, SF-36 scores, CO, age at intake) and outcomes (ie, CO-confirmed abstinence at 52-week follow-up) in the training set were normalised and scaled to have a mean of 0 and SD of 1. Numeric predictors in the testing set were normalised blindly using the same transformations as the training set.

## Results

### Participants

Smoking trajectories were modelled using a total of 5578 observations (weeks 2, 10, 19 and 26 had 1764, 1148, 1384 and 1282 observations, respectively) from 1783 participants.

### Descriptive data

Participants from five countries were 44.8% men (798/1783), with a mean (SD) age of 44.1 (10.7) years, smoked an average of 27.3 (9.7) CPD, had FTND scores of 6.1 (2.0) and started smoking at 16.9 (3.7) years of age (table 1). They had mean SF-36 scores (0=poor health, 100=better health) of 81.8 (30.7) for physical health, 81.7 (31.3) for emotional problems, 83.8 (20.6) for social functioning, 79.7 (22.8) for pain and 72.8 (16.5) for emotional well-being. At baseline, most (85.6%) reported more than two lifetime attempts to quit smoking, over half (56.6%) had attempted to quit within the year prior to trial enrolment and more than a third (38.7%) reported previously achieving over 3 months of abstinence during a prior quit attempt. We observed that a small number of participants (n=10) reported having never attempted to quit smoking. Results from a sensitivity analysis excluding these participants were similar to the primary findings and are reported in online supplemental materials.

**Table 1:** Participant characteristics by assigned latent class

	Full cohort	Analysis sample	Class 1	Class 2	Class 3
n (%)	2066	1783 (100)	186 (10.4)	803 (45.0)	794 (44.5)
Study site (%)					
Australia	436 (21.1)	360 (20.2)	32 (17.2)	159 (19.8)	169 (21.3)
Denmark	411 (19.9)	340 (19.1)	35 (18.8)	175 (21.8)	130 (16.4)
Germany	385 (18.6)	353 (19.8)	60 (32.3)	153 (19.1)	140 (17.6)
Switzerland	400 (19.4)	301 (16.9)	29 (15.6)	139 (17.3)	133 (16.8)
USA	434 (21.0)	429 (24.1)	30 (16.1)	177 (22.0)	222 (28.0)
Study Trt. Group=active (%)	1032 (50.0)	900 (50.5)	125 (67.2)	413 (51.4)	362 (45.6)
Sex=Male (%)	931 (45.1)	798 (44.8)	96 (51.6)	357 (44.5)	345 (43.5)
Age (mean (SD))	45.0 (10.8)	44.1 (10.7)	45.8 (11.4)	44.3 (10.5)	43.5 (10.7)
FTND (mean (SD))	6.1 (2.0)	6.1 (2.0)	5.6 (2.1)	6.1 (2.0)	6.3 (1.9)
CPD (mean (SD))	27.3 (9.7)	27.3 (9.7)	25.7 (10.4)	27.4 (9.8)	27.6 (9.5)
Motivation to quit (n (%))*					
Not at all	223 (11.1)	192 (11.1)	8 (4.3)	81 (10.4)	103 (13.4)
A little	701 (34.9)	596 (34.3)	58 (31.5)	270 (34.6)	268 (34.8)
Somewhat	496 (24.7)	435 (25.1)	49 (26.6)	185 (23.7)	201 (26.1)
A lot	589 (29.3)	513 (29.6)	69 (37.5)	245 (31.4)	199 (25.8)
Depression (n (%))					
Not at all	1675 (81.4)	1447 (81.2)	157 (84.4)	653 (81.3)	637 (80.2)
Somewhat	236 (11.5)	210 (11.8)	19 (10.2)	91 (11.3)	100 (12.6)
Moderately so	93 (4.5)	79 (4.4)	5 (2.7)	33 (4.1)	41 (5.2)
Very much so	46 (2.2)	40 (2.2)	4 (2.2)	23 (2.9)	13 (1.6)
Extremely so	8 (0.4)	7 (0.4)	1 (0.5)	3 (0.4)	3 (0.4)
Anxiety (n (%))					
Not at all	1515 (73.6)	1286 (72.1)	152 (81.7)	589 (73.3)	545 (68.6)
Somewhat	319 (15.5)	300 (16.8)	19 (10.2)	128 (15.9)	153 (19.3)
Moderately so	128 (6.2)	110 (6.2)	12 (6.5)	46 (5.7)	52 (6.5)
Very much so	75 (3.6)	68 (3.8)	2 (1.1)	30 (3.7)	36 (4.5)
Extremely so	21 (1.0)	19 (1.1)	1 (0.5)	10 (1.2)	8 (1.0)

* Though inclusion criteria required that participants did not have plans to quit smoking in the next month, baseline motivation to quit was assessed separately by asking ‘How much would you say you want to stop smoking?’.

CPD, cigarettes-per-day; FTND, Fagerström Test for Nicotine Dependence (1=low dependence, 8+=high dependence).

Most participants (81.4%) reported having no depression, and 73.6% reported no anxiety. Of the 1783 participants included in the analysis sample, 900 (50.5%) were assigned to receive active NRT in the analysed trials (table 1). Please see online supplemental sTable 1 for a complete listing of baseline characteristics.

### Analysis 1: trajectories in CPD over time

Latent class model-fit information appears in online supplemental sTable 2, suggesting that models using 3–6 classes are optimal. The 3-class model was selected to balance minimal information criteria with parsimony; the 4, 5 and 6-class models did not reveal more information about smoking trajectories. Distributions of CPD changes for each follow-up point and a model-fit curve are available in online supplemental sFigures 1 and 2, respectively.

**Figure 3:**
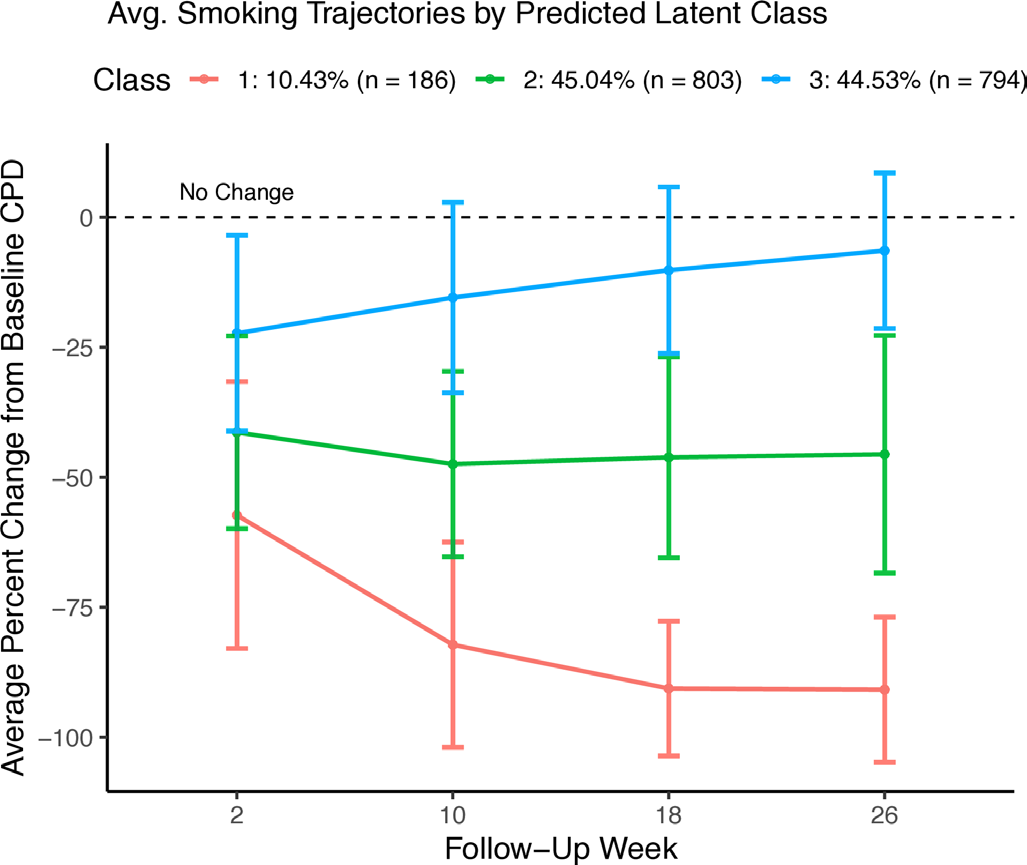
Average smoking trajectories for the 3-class model (ie, change from baseline smoking rate) (n=1783). Error bars represent SD from average CPD at each trial follow-up point. CPD, cigarettes-per-day.

Class 1 (n=186, 10.4%) is characterised by an average initial reduction in CPD of approximately 57% at week 2, followed by a trend toward 90% reduction by week 26. Participants in Class 2 (n=803, 45.0%) initially reduced their smoking by approximately 41% and remained consistent, for an average reduction of 46% from baseline at week 26. Class 3 participants (n=794, 44.5%), in contrast, showed a modest (approximately 22%) reduction in smoking and a return to near-baseline levels (ie, 6% reduction from baseline) by week 26. Average CPD trajectories for each of the selected three classes are shown in figure 3.

Fixed-effects maximum likelihood estimations show the initial smoking reduction (ie, change from baseline smoking at week 2) for participants in Class 1 was significantly greater than Class 3 (estimate (SE)=−1.31 (0.11), Wald=−12.32, p<0.001), but initial smoking reductions among Class 2 participants did not differ from those in Class 3 (estimate (SE)=0.03 (0.08), Wald=0.38, p=0.701). Changes in CPD from baseline (ie, fixed effects) were observed for each week (p’s <0.001).

To assess the possible impacts of excluding participants with missing baseline measures, an additional LCA was conducted with all participants who had baseline and at least one post-baseline CPD value (online supplemental sFigure 3), as well as patterns of missingness by latent group (online supplemental sFigure 4). These trajectories were similar to the primary results.

### Analysis 2: predicting longitudinal trajectories in CPD using baseline variables

Regularised logistic regression models predicting membership to Classes 1, 2 and 3 versus all performed better than chance (validation AUCs: Class 1=0.766, p<0.001; Class 2=0.569, p=0.008; Class 3=0.585, p<0.001), but membership to Class 2 versus all was closest to chance. The strongest predictive performance was seen predicting Class 1 versus Class 3 (AUC=0.788, p<0.001) and Class 1 versus Class 2 (AUC=0.784, p<0.001), while Class 2 versus Class 3 was near chance (AUC=0.523, p<0.001). See online supplemental sTable 3 for cross-validated AUC scores and online supplemental sFigure 5a for one-versus-all ROC curves.

**Figure 4:**
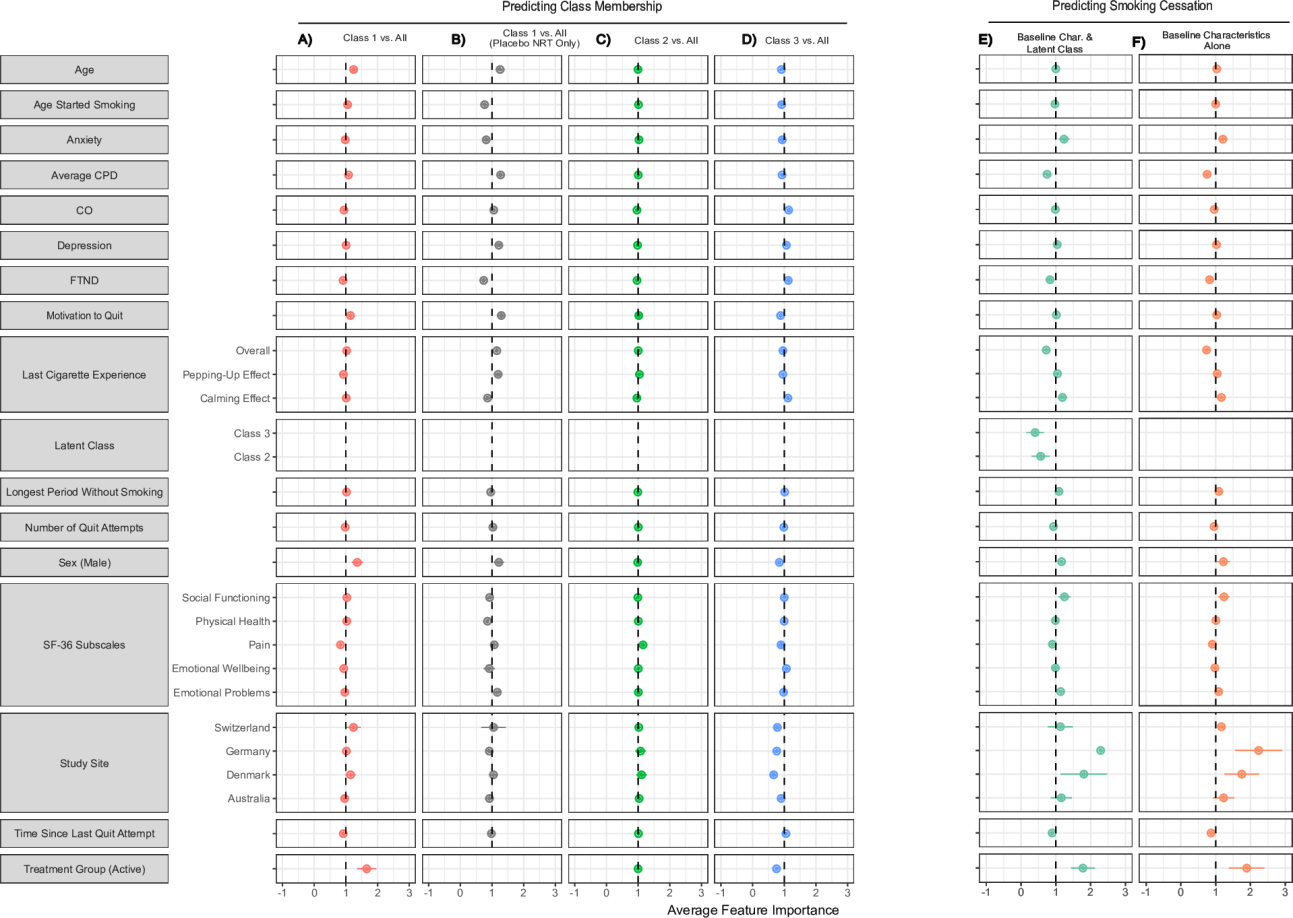
Feature importance from prediction of class membership using baseline characteristics (columns A–D) and smoking cessation 6 months following the trial (columns E and F). Each column represents a separate cross-validated binary logistic regression model. Values represent mean ORs for regularised binary logistic regression coefficients across five outer validation folds. Error bars represent the SD from these averages. Dashed lines represent ORs of 1, or no effect. Columns A–D: each class was predicted using one-versus-all classification. Values to the left of the dashed lines represent decreased odds of membership to a particular latent trajectory, while values to the right represent increased odds. Notably, participants in Class 1—those who reduced smoking the most—tended to be slightly older, score lower on anxiety symptoms at baseline and were more likely to be men. As expected, those in Class 1 were more likely to have received active NRT during the trial. In contrast, participants in Class 3 tended to have lower baseline CPD values, score lower on depression symptoms, were more likely to be women and to have higher baseline nicotine dependence scores. These participants were also more likely to have received placebo NRT during the trial. Column B: average feature importance for a binary logistic regression model using only the 900 (50.5%) who received placebo NRT, predicting membership to Class 1, the group which reduced their smoking the most. In this group, older participants presenting with lower anxiety scores, and those who have tried to quit before, were more likely to reduce their smoking. Columns E and F: values to the left of the dashed lines represent decreased odds of smoking cessation after the trial, while values to the right represent increased cessation odds. When using latent class as a predictor (left column), latent class becomes one of the predominant associations with smoking cessation (Class 2: OR=0.111±0.013, Class 3: OR=0.070±0.005), along with receiving active NRT (OR=1.90±0.157). A large site effect was observed, with participants at sites in Denmark and Germany more likely to quit smoking than those at the USA site. CO, carbon monoxide; CPD, cigarettes-per-day; FTND, Fagerström Test for Nicotine Dependence; NRT, nicotine replacement therapy; SF-36, Short Form Health Survey-36.

The relative contributions of each baseline characteristic to the overall model’s predictive capacity are presented as cross-validated averages in figure 4 (values are listed in online supplemental sTable 4). Participants assigned to Class 1—those who reduced smoking substantially—tended to be older and have lower levels of anxiety and nicotine dependence. They were more motivated to quit smoking at study baseline and tended to have slightly higher SF-36 social and physical sub-scores, but below average pain sub-scores. Intuitively, those in Class 1—the group which reduced its smoking the most—were more likely to have received active NRT, while those in Class 3 were more likely to have received placebo treatment. Although the greatest number of participants was assigned to Class 2, the model predicting membership to Class 2 showed low overall performance. Consequently, no distinct pattern of features describing Class 2 emerges.

Since the study intervention was not a clear predictor of latent class, a post hoc one-versus-all logistic regression model was used to predict membership to Class 1 within the 900 (50.5%) participants who received placebo NRT. These participants were more likely to be men, older at baseline, present with lower anxiety scores and were more likely to have tried quitting before.

Large site effects were observed. Relative to participants in the USA, participants at the other four sites (Switzerland, Germany, Denmark and Australia) were more likely to follow Class 1’s trajectory and less likely to follow Class 3’s. Additional predictive models were used to determine characteristic patterns associated with individual sites, and no distinct patterns emerged.

### Analysis 3: which trajectories in CPD predict smoking cessation?

Of the 1783 participants assigned to latent classes, 122 (6.8%) met criteria for biochemically verified smoking cessation (Class 1: 70/186 (37.6%); Class 2: 34/803 (4.2%); Class 3: 18/776 (2.3%)) at the 52-week follow-up (ie, approximately 50 weeks after the largest reductions in CPD for each class). Regularised logistic regression was used to predict smoking cessation using baseline characteristics alone (AUC=0.632±0.006, p<0.001), and baseline characteristics plus latent class (AUC=0.776±0.010, p<0.001). Each model outperformed classification using a permuted null distribution. Adding latent class as a predictor improved cessation prediction by 14.4%. See online supplemental sFigure 5b for ROC curves.

As with the previous analysis, regression coefficients from each model’s validation folds were recorded to assess feature importance in predicting smoking cessation (see figure 4; values are listed in online supplemental sTable 5). In the model using latent class as a predictor, participants in Classes 2 and 3 were approximately 90% less likely to achieve smoking cessation 6 months following the trial than Class 1 (Class 2 OR=0.111±0.013, Class 3 OR=0.070±0.005). Participants who received active NRT were also more likely to quit smoking (OR=1.90±0.157). A large site effect was observed, with participants at sites in Denmark and Germany more likely to quit smoking than those at the USA site. To address this, an additional model predicting follow-up cessation was fit without the USA site included, and the observed pattern of results remained similar (see online supplemental sFigure 6). Additionally, predictive models restricted to participants who had 52-week follow-up CO values were fit, and the pattern of results remained similar (see online supplemental sFigures 6–8).

## Discussion

This study examined smoking patterns in a secondary analysis of five NRT trials and found three distinct repeated measures smoking trajectories among participants who did not have immediate plans to quit smoking at baseline. Approximately 10% of participants initially reduced and nearly or completely eliminated their smoking (Class 1), 45% reduced by approximately half of their baseline CPD and remained at that level throughout the trial (Class 2) and the remaining 45% reduced initially but reverted to cigarette use similar to their baseline levels (Class 3).

Predictive modelling revealed that participants who reduced the most tended to be men, older and have lower levels of anxiety. This trend was emphasised when comparing participants who reduced despite receiving placebo NRT versus those who failed to reduce substantially yet received active NRT. Additionally, regression models using baseline characteristics plus latent class assignment to predict smoking cessation at 52 weeks follow-up outperformed models including baseline characteristics alone. This suggests that smoking trajectories, including initial patterns of reduction, may have implications for predicting smoking cessation outcomes.

LCA can help identify patterns in homogeneity in cigarette use which may not have been apparent from descriptive statistics or linear models alone. Most importantly, despite random assignment to active or placebo NRT and consistent instructions to reduce smoking, LCA reveals that cigarette use trajectories were not homogeneous. In particular, although those who received active NRT were more likely to reduce their smoking, many participants did not reduce. Lower levels of nicotine dependence and anxiety predicted reduction, even among those given placebo NRT.

Consistent with prior research,^41 42^ those who failed to reduce their smoking during the trial or who only cut out a small number of cigarettes were less likely to have quit smoking following the trial. Although reduction and cessation patterns aligned with treatment assignment, baseline reports of anxiety, social functioning and nicotine dependence were similarly important. In the same predictive model, latent class and random assignment to active or placebo NRT were both strongly associated with follow-up smoking cessation. This suggests that, in addition to pharmacotherapy, a focus on initial smoking reduction trajectory (ie, as few as 2 weeks following instructions to reduce) is important when considering who is likely to benefit from an intervention to reduce smoking among those not ready to quit abruptly, and who may need further support. Our findings also indicate that those with lower anxiety achieved greater reductions in CPD, suggesting interventions that address anxiety could help to maximise smoking reduction.

When asked to reduce smoking, those with substantial reductions after 2 weeks may follow paths toward substantial reduction or quitting, while those with minimal initial reductions may revert to their usual smoking levels. These results align with a previous LCA^43^ which concluded that people who smoke and are not ready to quit are heterogeneous.

### Directions for future research

The present study identified a substantial minority of people who were not motivated to quit smoking but reduced substantially when asked to. That subset was far more likely to achieve complete cessation, suggesting that those who reduce smoking successfully may be more likely to quit. However, it remains unclear whether this initial reduction causes cessation or if those able to reduce are also more likely to achieve complete cessation. Additionally, the rate of cessation at the 52-week follow-up in the analysed trials was relatively low (<7%), and the latent class most predictive of cessation only comprised 10% of the sample. Future research is needed to investigate adaptive interventions wherein a targeted intervention is provided to improve outcomes among individuals who do not initially respond to reduction treatment. Furthermore, given the trials analysed in this study only included participants who were not ready to quit at baseline, an important next step would be to examine pre-cessation smoking behaviour in a sample of people who are trying to quit smoking.

### Strengths and limitations

This study had the following strengths: use of a large sample across multiple countries, using ML-based predictive modelling to find robust associations, and objective, information-theoretic approaches to selecting LCA models. Additionally, associations with smoking cessation benefit from biochemical verification rather than relying solely on self-report.

Limitations include that latent classes were developed based on self-reported CPD. Although it is possible that participants inaccurately reported their CPD, it was not possible to biochemically confirm CPD using CO. Those who reduce their CPD may inhale their remaining cigarettes more deeply, which could mean that the CO reading does not decrease in line with the reduction in CPD.^44^ Furthermore, CO readings are affected by the recency of smoking.^27^ Therefore, even if a person has reduced their daily CPD overall, this would not be reflected in the CO measurement if they had smoked their remaining cigarettes just before the measure was taken. Additionally, missing data could confound results, although imputation was minimal and applied within relevant groups to minimise bias. Although the homogeneity in methods across the analysed trials offers strong internal consistency, it may limit the generalisability of our findings to patients who do plan to quit smoking, use different NRT or receive more intensive behavioural treatment. We also note that data for these trials were collected over 20 years ago and therefore interpretation is limited given the changing demographics and behaviours of adults who smoke cigarettes and the evolving tobacco marketplace, including the introduction of alternative nicotine delivery systems.

### Conclusion

Our examination of latent trajectories in smoking behaviour among a sample of people who were not motivated to quit revealed heterogeneity in smoking patterns. Specifically, three distinct smoking trajectories were identified, with one nearly twice as likely as the others to achieve subsequent smoking cessation. These findings establish that smoking reduction by 50% or more is associated with a substantially increased likelihood of smoking cessation among people who were not ready to quit at baseline and demonstrate the importance of reduction during the first 2 weeks of a smoking intervention.

## Supplementary material

10.1136/bmjph-2024-001605 online supplemental file 1

## Data Availability

Data are available upon reasonable request.
