# Supplementary material for: Smoking reduction trajectories and their association with smoking cessation: a secondary analysis of longitudinal clinical trial data
Source: BMJ Public Health. 2025 Dec 25;3(2):e001605. doi: 10.1136/bmjph-2024-001605 (PMC12742168; doi:10.1136/bmjph-2024-001605)
Supplement: online supplemental file 1 [file bmjph-3-2-s001.docx]

Supplement to:

Smoking reduction trajectories and their association with smoking cessation: A secondary analysis of longitudinal RCT data

Anthony Barrows, MS, Elias Klemperer, PhD, Hugh Garavan, PhD, Nicholas Allgaier, PhD, Nicola Lindson, PhD, Gemma Taylor, PhD

## Latent Class Analysis Model Fit Information

Smoking trajectories were fit to percent change in cigarettes per day (CPD) from baseline using the distributions shown in sFigure 1 for each trial follow-up point except Week 52, which was reserved for smoking cessation prediction.


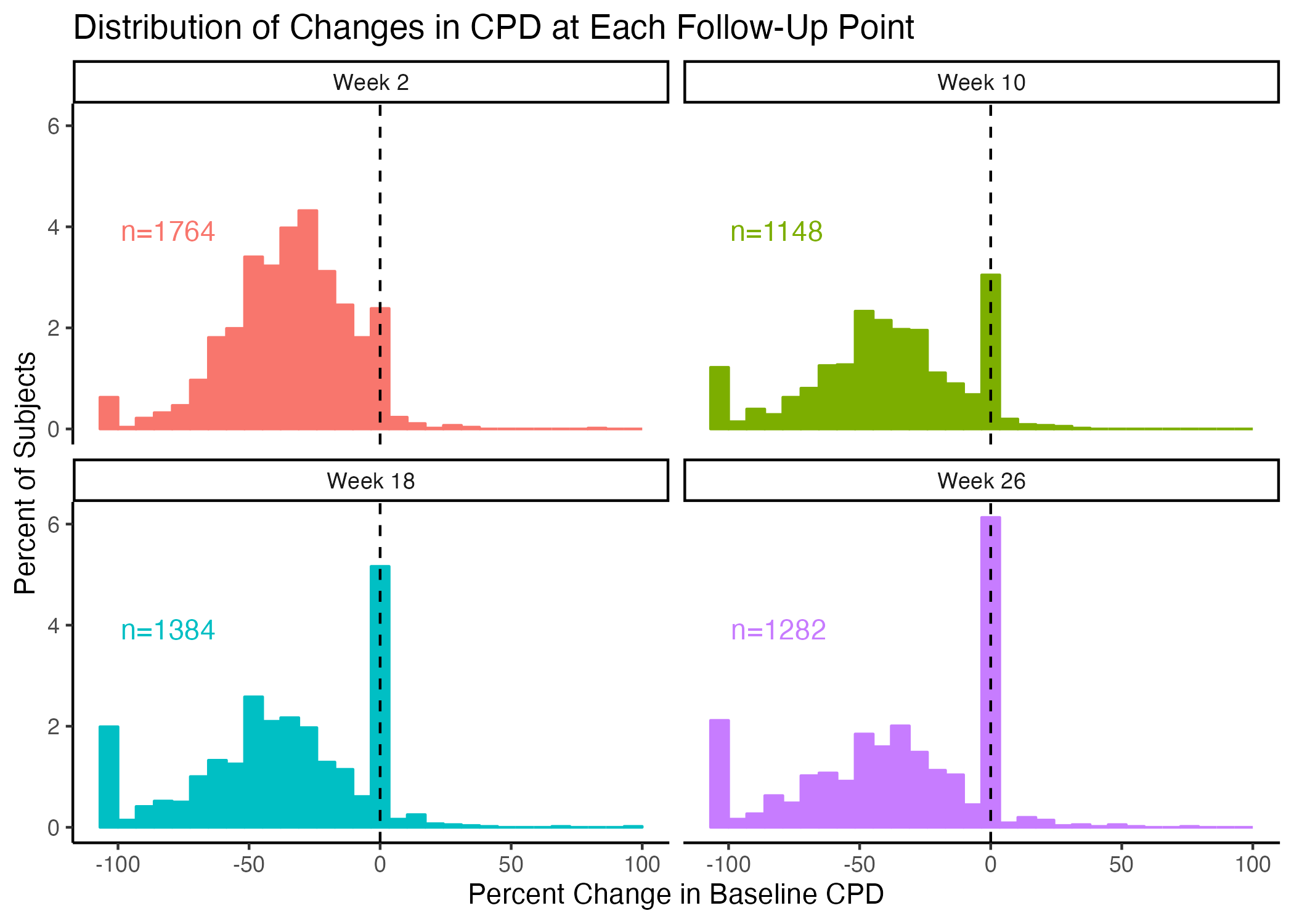


**sFigure 1.** Distributions of changes in cigarettes per day (CPD) as a percentage of baseline smoking rates (Total N = 1783). A value of 0 represent no change in smoking rate from baseline.


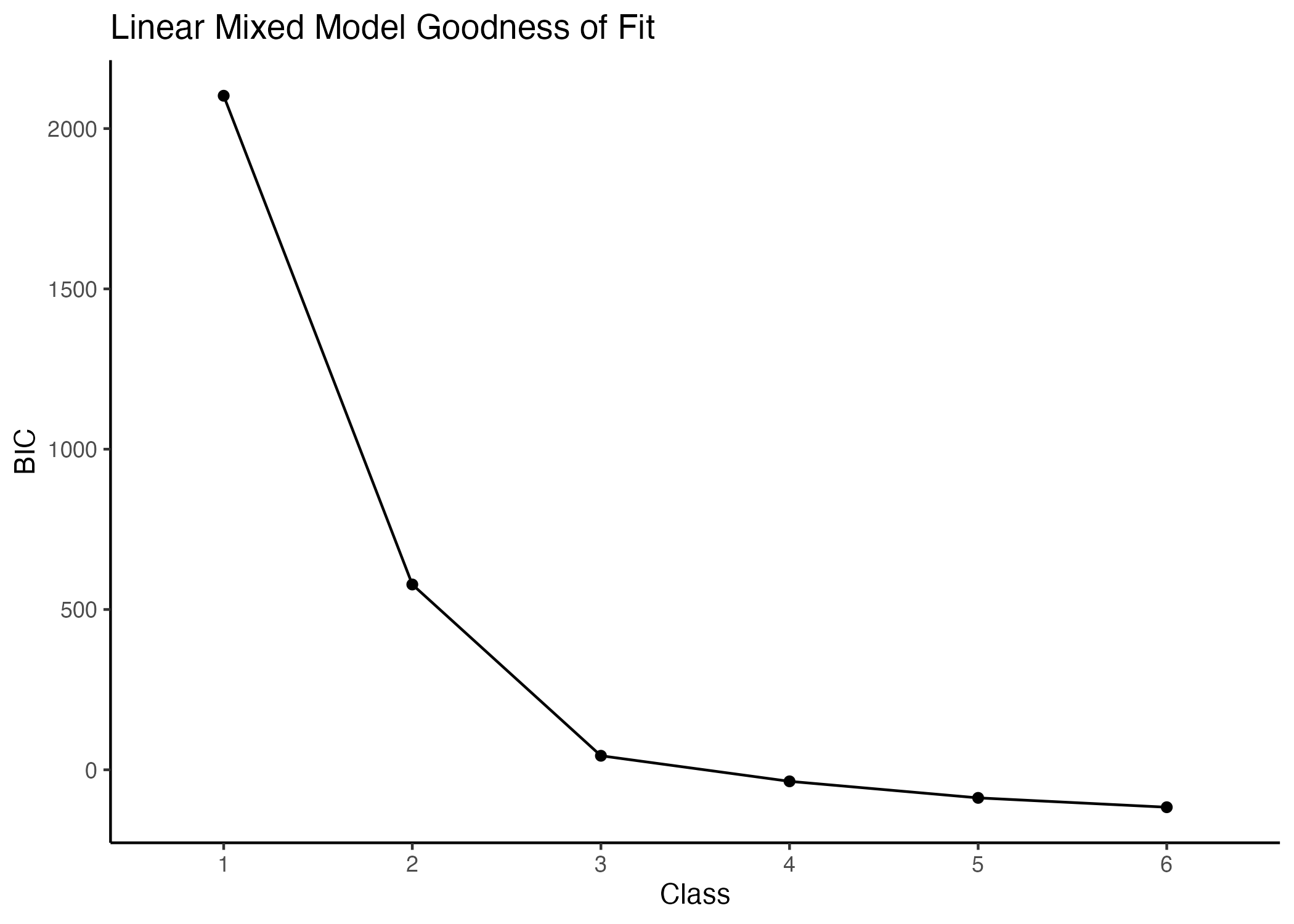


**sFigure 2**. Latent class mixture model BIC curve (n = 178)

Participants are assigned to latent classes using Bayes’ theorem, where class-memberships probabilities $\pi_{ig}$ for each participant $i \left( i=1,\ldots,N \right)$ for latent class $g\left( g=1,\ldots,G \right)$) is the probability of belonging to a particular class given the model parameters, estimated from the data:

$$\pi_{ig}=P\left( c_{i}=g | X_{i}, Y_{i,} \hat{\theta}_{G} \right)$$

where $c_{i}$ is a latent categorical variable (of latent classes $g\left( g=1,\ldots,G \right)$) given covariates $X_{i}$, outcome $Y_{i}$, and latent class model parameters $\hat{\theta}$ (Proust-Lima et al., 2014).

## Updated Smoking Cessation Verification Guidelines

Using <=11ppm CO as the threshold for biochemically verified smoking cessation, 135/1784 (7.5%) meet abstinence criteria (40.8% of Class 1, 4.7% of Class 2, and 2.6% of Class 3), compared with 122/1784 (6.8%) using <6ppm CO (37.6% of Class 1, 4.2% of Class 2, and 2.3% of Class 3. Using <11ppm CO as a verification threshold, participants in Classes 2 and 3 were substantially less likely to achieve smoking cessation 6 months following the trial compared to those in Class 1 (Class 2 OR = 0.112 ± 0.057, Class 3 OR = 0.066 ± 0.007).

Given the relatively small impact of reducing the verification threshold, we used <6ppm for smoking cessation predictive modeling, which is consistent with the most recent guidance.(1)

## Variable Coding

| Name | | | Type | Values |
| --- | --- | --- | --- | --- |
| Age | | | Numeric | Years |
| Sex | | | Binary | - Male - Female |
| Age Started Smoking | | | Numeric | Years |
| Carbon Monoxide (CO) | | | Numeric | Parts Per Million (ppm) |
| Cigarettes Per Day (CPD) | | | Numeric | Self-report Average |
| Fagerstrom Test for Nicotine Dependence | | | Numeric | Summary Score (0-10) |
| Intention to Quit* | | | Ordinal | - Not at all - A little - Somewhat - A lot |
| Last Cigarette Experience (Relief from Smoking Questionnaire) | | Overall | Ordinal | - Very unpleasant - Somewhat unpleasant - Neutral - Somewhat pleasant - Very pleasant |
|  |  | Pepping-Up Effect |  |  |
|  |  | Calming Effect |  |  |
| Longest Period Without Smoking | | | Ordinal | - <1 week - 1 week – 1 month - >1 month – 3 months - >3 months |
| Number of Quit Attempts | | | Ordinal | - Never - Once - 2 to 5 times - 6 to 10 times - More than 10 times |
| Time Since Last Quit Attempt | | | Ordinal | - Never quit - >12 months - >12-24 months - >6-12 months - 0-6 months |
| Smoking Cessation Quality of Life (SCQoL) | Anxiety | | Ordinal | - Not at all - Somewhat - Moderately so - Very much so - Extremely so |
|  | Depression | |  |  |
| RAND 36-Item short-form survey | | Emotional Problems | Numeric | See (2) |
|  |  | Emotional Wellbeing |  |  |
|  |  | Pain |  |  |
|  |  | Physical Health |  |  |
|  |  | Social Functioning |  |  |
| Study Site | |  | Categorical | - Australia - Denmark - Germany - Switzerland - USA |
| Treatment Group | |  | Binary | - Active - Placebo |

*Four of the five included trials captured intention to quit smoking using continuous variables (i.e., “Rate the likelihood that you will quit smoking in the next 6 months,” or “How motivated are you to quit smoking?”), recorded from 0 to 10. In those cases, the continuous scales divided evenly into the four categories described above.

# Supplemental Tables

**sTable 1** Additional participant characteristics

|  | Full Cohort | Analysis Sample | Class 1 | Class 2 | Class 3 |
| --- | --- | --- | --- | --- | --- |
|  | 2066 | 1783 (100) | 186 (10.4) | 803 (45.0) | 794 (44.5) |
| SF-36 Scales |  |  |  |  |  |
| Physical Health | 81.8 (30.8) | 81.7 (30.8) | 82.9 (32.2) | 82.2 (30.9) | 81.0 (30.5) |
| Emotional Problems | 81.7 (31.3) | 81.7 (31.3) | 84.6 (29.2) | 80.9 (31.8) | 81.7 (31.4) |
| Social Functioning | 83.8 (20.6) | 83.8 (20.6) | 85.0 (20.3) | 84.1 (20.2) | 83.1 (21.2) |
| Pain | 79.7 (22.8) | 79.7 (22.8) | 79.2 (22.8) | 81.1 (23.1) | 78.4 (22.5) |
| Emotional Wellbeing | 72.8 (16.5) | 72.8 (16.5) | 73.1 (17.7) | 72.9 (16.5) | 72.6 (16.3) |
| Longest Period Without  Smoking (n (%)) |  |  |  |  |  |
| < 1 week | 465 (22.6) | 398 (22.3) | 30 (16.1) | 184 (22.9) | 184 (23.2) |
| 1 week – 1 month | 445 (21.6) | 390 (21.9) | 38 (20.4) | 166 (20.7) | 186 (23.4) |
| > 1month – 3 months | 351 (17.1) | 316 (17.7) | 35 (18.8) | 144 (17.9) | 137 (17.3) |
| > 3 months | 797 (38.7) | 679 (38.1) | 83 (44.6) | 309 (38.5) | 287 (36.1) |
| Age Started Smoking (mean (SD)) | 16.9 (3.8) | 16.9 (3.7) | 17.6 (4.4) | 17.1 (3.9) | 16.7 (3.4) |
| Num. Quit Attempts (n (%))* |  |  |  |  |  |
| Never Quit | 13 (0.6) | 10 (0.6) | 0 (0.0) | 4 (0.5) | 6 (0.8) |
| 1 | 285 (13.8) | 238 (13.3) | 31 (16.7) | 110 (13.7) | 97 (12.2) |
| 2 – 5 | 1337 (65.0) | 1164 (65.3) | 114 (61.3) | 516 (64.3) | 534 (67.3) |
| 6 – 10 | 273 (13.3) | 244 (13.7) | 28 (15.1) | 112 (13.9) | 104 (13.1) |
| > 10 | 150 (7.3) | 127 (7.1) | 13 (7.0) | 61 (7.6) | 53 (6.7) |
| Time Since Last Quit Attempt (n (%) |  |  |  |  |  |
| Never Quit | 13 (0.6) | 10 (0.6) | 0 (0.0) | 4 (0.5) | 6 (0.8) |
| > 24 Months | 128 (6.2) | 128 (7.2) | 13 (7.0) | 47 (5.9) | 68 (8.6) |
| 12-24 Months | 752 (36.5) | 644 (36.1) | 77 (41.4) | 298 (37.1) | 269 (33.9) |
| 6-12 Months | 693 (33.7) | 580 (32.5) | 59 (31.7) | 263 (32.8) | 258 (32.5) |
| 0-6 Months | 472 (22.9) | 421 (23.6) | 37 (19.9) | 191 (23.8) | 193 (24.3) |
| RSQ Last Cigarette Experience (n (%)) |  |  |  |  |  |
| Very Unpleasant | 44 (2.1) | 39 (2.2) | 3 (1.6) | 14 (1.7) | 22 (2.8) |
| Somewhat Unpleasant | 202 (9.8) | 168 (9.4) | 21 (11.3) | 84 (10.5) | 63 (7.9) |
| Neutral | 787 (38.3) | 688 (38.6) | 65 (34.9) | 306 (38.2) | 317 (39.9) |
| Somewhat Pleasant | 638 (31.0) | 564 (31.6) | 70 (37.6) | 255 (31.8) | 239 (30.1) |
| Very Pleasant | 386 (18.8) | 323 (18.1) | 27 (14.5) | 143 (17.8) | 153 (19.3) |
| RSQ Calming Effect (mean (SD)) | 4.7 (1.8) | 4.6 (1.8) | 4.5 (1.8) | 4.6 (1.8) | 4.7 (1.7) |
| RSQ Pepping-Up Effect (mean (SD)) | 4.0 (2.3) | 3.8 (2.3) | 3.7 (2.3) | 3.9 (2.3) | 3.9 (2.2) |

SF-36 = RAND 36-Item Health Survey (0 = poor health, 100 = better health). RSQ = Relief from Smoking Questionnaire; for RSQ Calming Effect and Pepping-Up Effect scales 0 = not at all, 4 = very strong.

*We conducted sensitivity analyses excluding the 10 participants whose self-reported baseline data conflicted with exclusion criteria (i.e., at least one quit attempt). Results from these analyses were similar to primary findings in direction and significance (reported later in this Supplement).

**sTable 2** Latent class model fit comparison (n = 1783). *Selected model. BIC = Bayesian Information Criterion, AIC = Akaike Information Criterion.

| Number of Classes | BIC | AIC | Maximum Log-Likelihood |
| --- | --- | --- | --- |
| 1 | 2102.42 | 2085.97 | -1039.98 |
| 2 | 577.95 | 545.04 | -266.52 |
| 3* | 43.75 | -5.62 | 11.81 |
| 4 | -36.02 | -101.85 | 62.92 |
| 5 | -87.61 | -169.90 | 99.95 |
| 6 | -116.79 | -215.54 | 125.77 |

**sTable 3** *Average AUC across five-fold cross-validation $\text{±}$ SEM. **AUC using unseen data. p values represent the probability that the test AUC value is greater than the average computed null AUC value for the test data.

|  | Mean CV AUC* | Test AUC** | $\text{p}$ |
| --- | --- | --- | --- |
| Class 1 vs. All | $\text{0.6}\text{57}\text{±0.0}\text{27}$ | 0.766 | $\text{<.001}$ |
| Class 2 vs. All | $\text{0.5}\text{35}\text{±0.01}\text{5}$ | 0.569 | $\text{.008}$ |
| Class 3 vs. All | $\text{0.5}\text{67}\text{±0.020}$ | 0.585 | $\text{<.001}$ |
| Class 1 vs. Class 2 | $\text{0.5}\text{70}\text{±0.0}\text{30}$ | 0.784 | $\text{<.001}$ |
| Class 1 vs. Class 3 | $\text{0.6}\text{70}\text{±0.0}\text{47}$ | 0.788 | $\text{<.001}$ |
| Class 2 vs. Class 3 | $\text{0.5}\text{30}\text{±0.0}\text{10}$ | 0.523 | $\text{<.001}$ |
| Class 1 vs. All (Placebo NRT Only) | $\text{0.6}\text{10}\text{±0.0}\text{46}$ | 0.717 | $\text{<.001}$ |

|  |  |  |  |  |
| --- | --- | --- | --- | --- |
|  |  |  |  |  |
|  |  |  |  |  |
|  |  | |  |  |

**sTable 4** Class prediction coefficients (means and standard deviations from cross-validated Elastic Net logistic regression) expressed as odds ratios.

| **Feature** | **Level** | **Class 1 vs.**  **All (mean)** | **Class 1 vs.**  **All (std)** | **Class 2 vs.**  **All (mean)** | **Class 2 vs.**  **All (std)** | **Class 3 vs.**  **All (mean)** | **Class 3 vs.**  **All (std)** | **Class 1 vs. All (Placebo NRT Only) (mean)** | | **Class 1 vs. All (Placebo NRT Only) (std)** |
| --- | --- | --- | --- | --- | --- | --- | --- | --- | --- | --- |
| Intercept |  | 0.107 | 0.024 | 0.781 | 0.042 | 1.012 | 0.094 | | 0.11 | 0.013 |
| Age |  | 1.244 | 0.077 | 0.995 | 0.01 | 0.909 | 0.023 | | 1.257 | 0.145 |
| Age Started Smoking | | 1.056 | 0.053 | 1.011 | 0.016 | 0.919 | 0.033 | | 0.766 | 0.079 |
| Anxiety |  | 0.982 | 0.047 | 1.026 | 0.058 | 0.936 | 0.04 | | 0.818 | 0.084 |
| Average CPD | | 1.084 | 0.062 | 1.003 | 0.008 | 0.93 | 0.032 | | 1.272 | 0.129 |
| CO |  | 0.943 | 0.045 | 0.961 | 0.041 | 1.138 | 0.042 | | 1.058 | 0.077 |
| Depression |  | 1.008 | 0.062 | 0.981 | 0.028 | 1.066 | 0.042 | | 1.215 | 0.091 |
| FTND |  | 0.912 | 0.059 | 0.964 | 0.052 | 1.125 | 0.056 | | 0.739 | 0.053 |
| Intention to Quit | | 1.145 | 0.068 | 1.019 | 0.043 | 0.882 | 0.043 | | 1.293 | 0.116 |
| Longest Period Without Smoking | | 1.022 | 0.071 | 0.99 | 0.023 | 1.014 | 0.029 | | 0.959 | 0.056 |
| Number of Quit Attempts | | 0.98 | 0.038 | 1.004 | 0.01 | 0.986 | 0.044 | | 1.029 | 0.075 |
| Last Cigarette Experience | Calming Effect | 1.013 | 0.105 | 0.96 | 0.089 | 1.115 | 0.062 | | 0.86 | 0.128 |
| Last Cigarette Experience | Overall | 1.026 | 0.044 | 1.004 | 0.009 | 0.958 | 0.022 | | 1.152 | 0.051 |
| Last Cigarette Experience | Pepping-Up Effect | 0.924 | 0.097 | 1.042 | 0.095 | 0.956 | 0.025 | | 1.194 | 0.13 |
| Sex (Male) |  | 1.363 | 0.176 | 0.984 | 0.036 | 0.844 | 0.051 | | 1.216 | 0.159 |
| SF-36 Subscales | Emotional Problems | 0.968 | 0.01 | 1.007 | 0.016 | 0.978 | 0.015 | | 1.167 | 0.097 |
| SF-36 Subscales | Emotional Wellbeing | 0.934 | 0.052 | 1.004 | 0.01 | 1.065 | 0.038 | | 0.913 | 0.176 |
| SF-36 Subscales | Pain | 0.832 | 0.055 | 1.154 | 0.071 | 0.895 | 0.044 | | 1.07 | 0.078 |
| SF-36 Subscales | Physical Health | 1.029 | 0.036 | 1.005 | 0.011 | 0.997 | 0.02 | | 0.859 | 0.049 |
| SF-36 Subscales | Social Functioning | 1.033 | 0.025 | 0.991 | 0.021 | 0.999 | 0.047 | | 0.925 | 0.107 |
| Study Site | Australia | 0.964 | 0.076 | 1.032 | 0.071 | 0.9 | 0.065 | | 0.921 | 0.146 |
| Study Site | Denmark | 1.149 | 0.164 | 1.109 | 0.16 | 0.664 | 0.074 | | 1.046 | 0.143 |
| Study Site | Germany | 1.017 | 0.065 | 1.077 | 0.168 | 0.763 | 0.101 | | 0.914 | 0.119 |
| Study Site | Switzerland | 1.239 | 0.231 | 1.021 | 0.046 | 0.78 | 0.129 | | 1.049 | 0.386 |
| Treatment Group (Active) | | 1.658 | 0.302 | 0.997 | 0.006 | 0.753 | 0.022 | | NA | NA |
| Time Since Last Quit Attempt | | 0.92 | 0.037 | 1.01 | 0.021 | 1.053 | 0.051 | | 0.98 | 0.08 |

**sTable 5** 1-year cessation prediction coefficients (means and standard deviations from cross-validated Elastic Net logistic regression) expressed as odds ratios.

| **Feature** | **Level** | **Baseline Char. & Latent Class (mean)** | **Baseline Char. & Latent Class (std)** | **Baseline Characteristics  Alone (mean)** | **Baseline Characteristics  Alone (std)** |
| --- | --- | --- | --- | --- | --- |
| Age |  | 0.998 | 0.042 | 1.031 | 0.079 |
| Age Started Smoking | | 0.974 | 0.066 | 1.002 | 0.047 |
| Anxiety |  | 1.235 | 0.153 | 1.205 | 0.123 |
| Average CPD | | 0.747 | 0.085 | 0.752 | 0.098 |
| CO |  | 0.986 | 0.027 | 0.96 | 0.067 |
| Depression |  | 1.039 | 0.093 | 1.024 | 0.045 |
| FTND |  | 0.833 | 0.06 | 0.825 | 0.054 |
| Intention to Quit | | 1.015 | 0.056 | 1.03 | 0.081 |
| Intercept |  | 0.014 | 0.025 | 0.016 | 0.023 |
| Last Cigarette Experience | Calming Effect | 1.19 | 0.132 | 1.162 | 0.136 |
| Last Cigarette Experience | Pepping-Up Effect | 1.046 | 0.06 | 1.044 | 0.043 |
| Last Cigarette Experience | Overall | 0.725 | 0.107 | 0.737 | 0.111 |
| Latent Class | Class 2 | 0.563 | 0.256 | NA | NA |
| Latent Class | Class 3 | 0.404 | 0.249 | NA | NA |
| Longest Period Without Smoking | | 1.09 | 0.077 | 1.09 | 0.094 |
| Number of Quit Attempts | | 0.931 | 0.04 | 0.952 | 0.056 |
| Sex (Male) |  | 1.165 | 0.095 | 1.221 | 0.189 |
| SF-36 Subscales | Emotional Problems | 1.138 | 0.111 | 1.085 | 0.097 |
| SF-36 Subscales | Emotional Wellbeing | 0.988 | 0.102 | 0.978 | 0.069 |
| SF-36 Subscales | Pain | 0.902 | 0.082 | 0.904 | 0.077 |
| SF-36 Subscales | Physical Health | 0.988 | 0.071 | 1.008 | 0.049 |
| SF-36 Subscales | Social Functioning | 1.247 | 0.171 | 1.238 | 0.159 |
| Study Site | Australia | 1.158 | 0.304 | 1.228 | 0.305 |
| Study Site | Denmark | 1.805 | 0.672 | 1.749 | 0.499 |
| Study Site | Germany | 2.287 | 0.763 | 2.233 | 0.677 |
| Study Site | Switzerland | 1.131 | 0.359 | 1.164 | 0.101 |
| Time Since Last Quit Attempt | | 0.894 | 0.06 | 0.867 | 0.071 |
| Treatment Group (Active) | | 1.778 | 0.341 | 1.891 | 0.51 |

# New Trajectory Model Using All Participants

The model included in the main text is restricted to participants without large patterns of missing data. To examine whether these patterns of missingness confound the latent class analysis, we fit a three-class model using the 1,884 subjects who had baseline and at least one other CPD values. Class assignments show similar trajectories in CPD across time points to those in the model using a restricted sample (see sFigure 4).


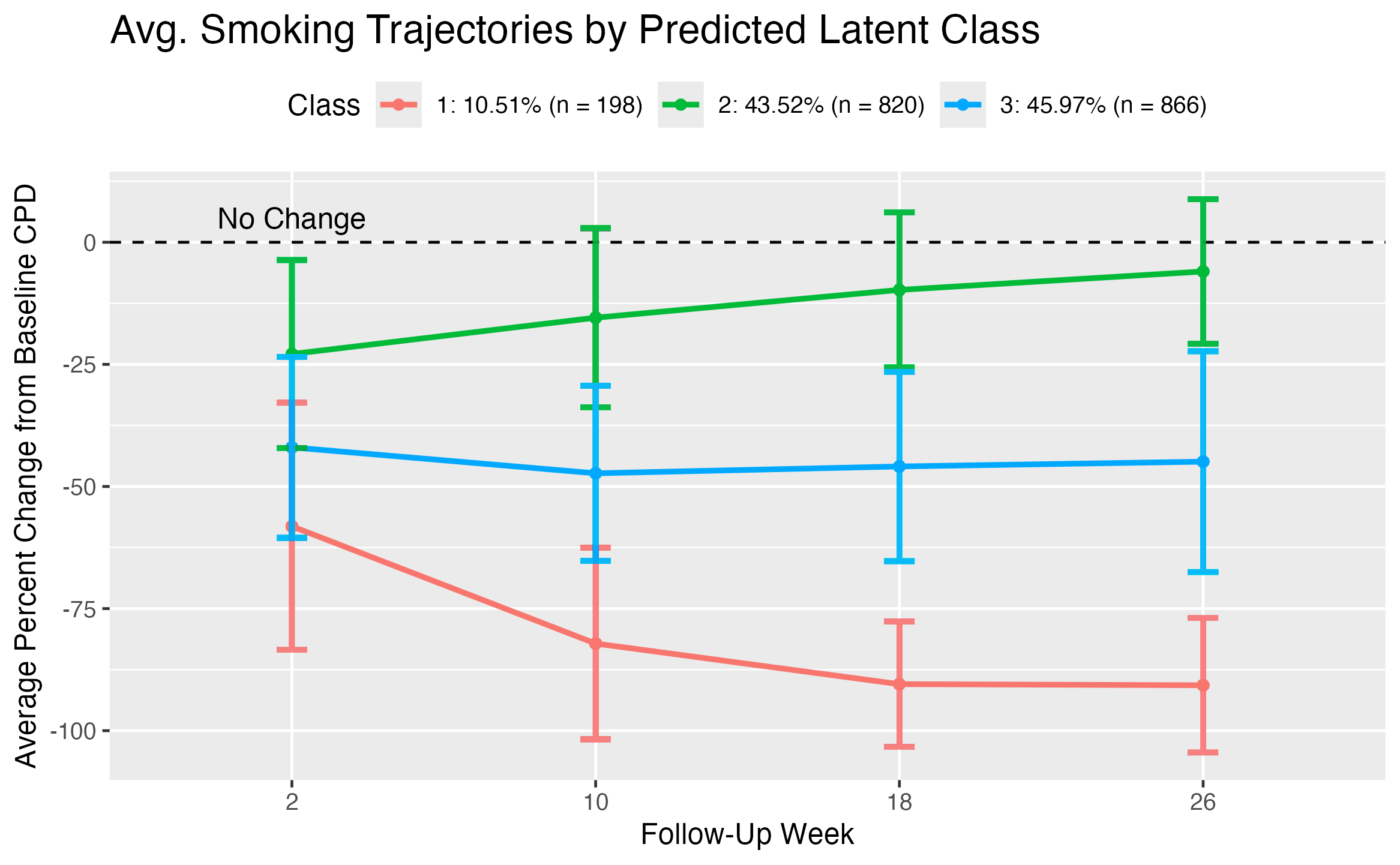


**sFigure 3** Latent class model fit before removing missing data.

sFigure 4 shows an informal breakdown of missingness by class. As the reviewer correctly identified, the SF-36 variables exhibited the greatest missingness. Limiting the dataset to just the SF-36 variables included in this work (i.e., Social Functioning, Physical Health, Pain, General, Emotional Wellbeing, and Emotional Problems), analysis of variance (ANOVA) shows suggestive evidence of group differences (F(df) = 2.993, p=.0504), but post-hoc pairwise comparisons (Tukey’s HSD) shows no differences between classes 2 and 1 (95% C.I. for difference = [-0.039, 0.039], p > .999), between 3 and 1 (95% C.I = [-0.014, 0.062], p > .326), or between 3 and 2 (95% C.I = [-0.000, 0.048], p > 0.052). We are therefore confident that group differences are unlikely to be due to missingness.

#
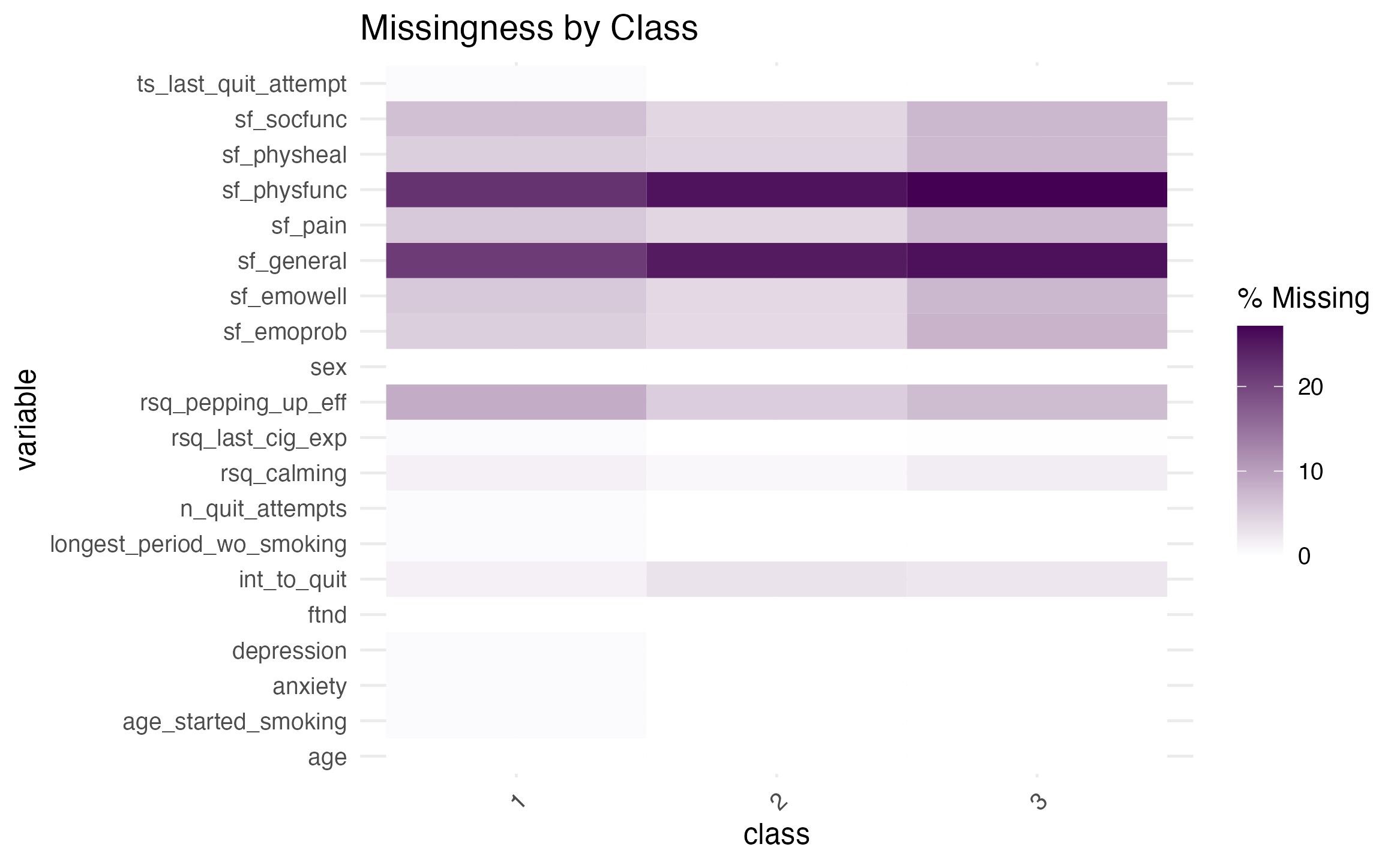


**sFigure 4** Predictor missingness by latent class for a latent class model fit to the full 1,884 participants with baseline and at least one subsequent CPD value recorded.

# Predictive Model Goodness of Fit and Hyperparameter Selection


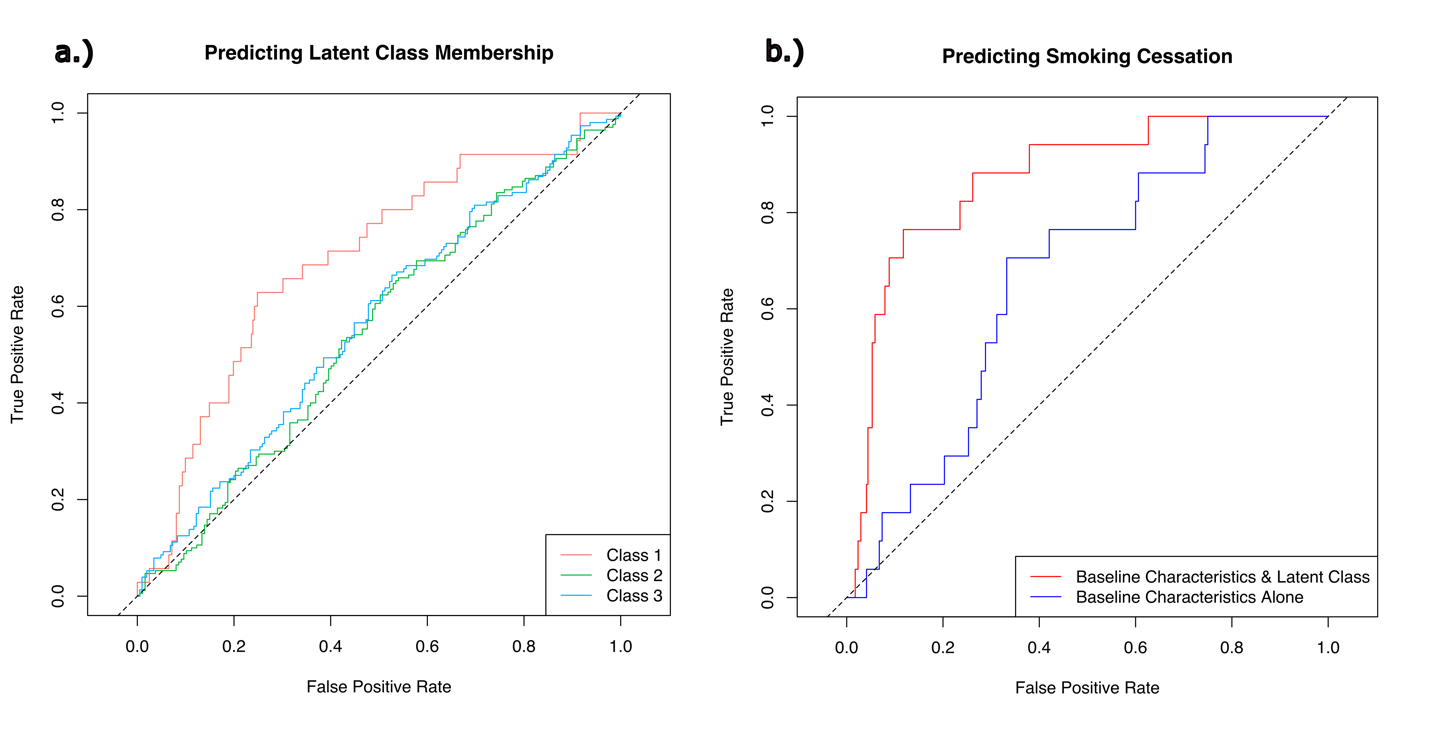


**sFigure 5.** Receiver operating characteristic (ROC) curves for regularized binary logistic regression predictive models. **a.)** Prediction of smoking trajectories as a proportion of baseline smoking during the trial. Each curve represents one-versus-all prediction to latent trajectories 1, 2, and, 3 in turn. The model predicting membership to Class 1, the group who reduced the most, performed best (AUC = 0.657 ± 0.027). All three models performed better than classification using a permuted null distribution (p’s <.001). **b.)** Smoking cessation prediction 6 months after the trial using participant baseline characteristics alone (blue) and baseline characteristics plus latent class as a predictor (red). Adding latent class as a predictor improved classification performance by an average of 14.4% (AUC = 0.776 ± 0.010, p = 0.002), suggesting smoking trajectories among people not looking to quit may be meaningful for long-term cessation outcomes. Each model performed better than classification using a permuted null distribution (p’s <.001).

Ten values evenly spaced on a log scale between approximately 10^-10^ and 1 were selected for each elastic net logistic regression hyperparameter (i.e., lambda, which controls the overall regularization strength, and alpha, which controls the mix between lasso and ridge regularization penalties; these are referred to as “penalty” and “mixture” parameters respectively in the glmnet engine used in this analysis (Friedman et al., 2010)). Larger values of lambda (i.e., stronger regularization overall) are likely to result in a simpler model, but with the risk of underfitting, whereas smaller values tend to lead to more complex models with the risk of overfitting. Larger values of alpha (i.e., more lasso) emphasize predictor sparsity, whereas smaller values (i.e., more ridge) retains all predictors by shrinking all coefficients (Zou & Hastie, 2005).

Models were fit using a nested cross validation scheme, in which the training set was randomly partitioned into five model training and model evaluation sets. Each model training partition was then randomly partitioned into 10 hyperparameter selection and evaluation sets. Within these partitions, models using all 100 combinations of alpha and lambda were evaluated, and the hyperparameter combination yielding the optimal ROC AUC was chosen for that validation fold and evaluated on the model evaluation set.

# New Cessation Prediction Model without USA Trial

In the prediction of smoking cessation at the 1-year follow up point, where those in the Germany trial, for example, were more than twice as likely to have achieved cessation as those in the USA trial. Using latent class assignment as a predictor still improves prediction of follow-up cessation substantially (i.e., by approximately 18%; AUC with latent class = $0.771 \pm0.013$ versus $0.584 \pm0.006$ without). sFigure 6 shows average coefficients from these models (presented as odds ratios). Although a site effect remains (participants from the Denmark trial were nearly twice as likely to achieve cessation compared to, in this supplementary analysis, those in the Australia trial), latent class assignment remains a strong predictor of cessation. Specifically, those who reduced their smoking substantially within the first two weeks of the intervention and continued reducing were more than twice as likely to achieve cessation as those who reduced their smoking but maintained that level (Class 2 odds ratio = $0.240 \pm0.263$, ref: Class 1) or those who reduced minimally and reverted to their baseline levels (Class 3 odds ratio = $0.165 \pm0.226$, ref: Class 1).


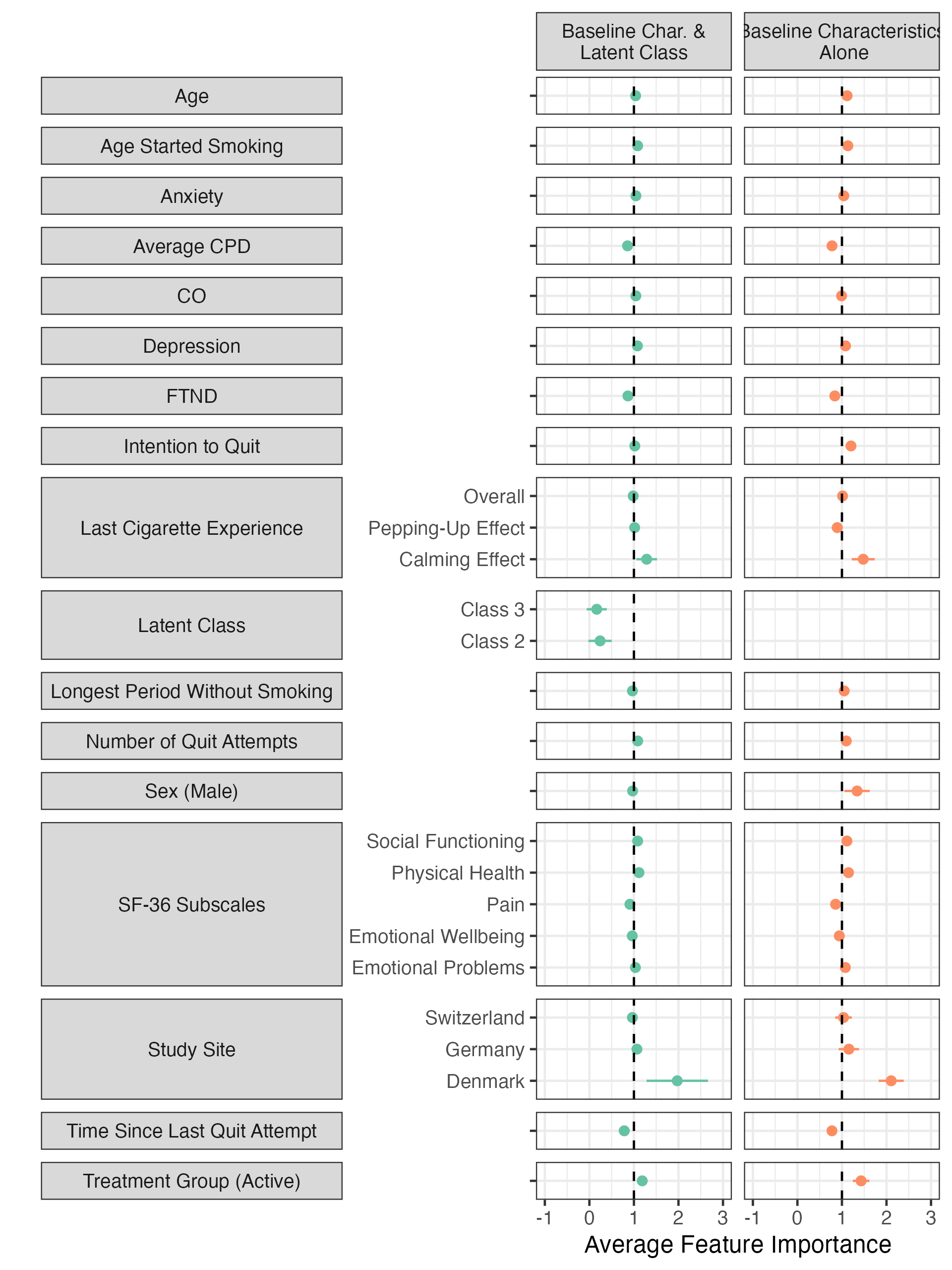


**sFigure 6** Follow-up (1-year) cessation prediction without US trial included to address site effect (reference for the “Study Site” predictor is now the Australian trial (n=360)). Feature importances are presented as odds ratios.

# New Predictive Models with CO-Only Sample

There is a large degree of missingness in 1-year follow-up CO values used to verify smoking cessation. In order to examine whether this missingness confounded cessation prediction, we limited the post-latent-class-assignment sample to those with recorded CO values at the 1-year follow-up point and repeated the cross-validated predictive modeling.

This restricted sample (Class 1 n = 143; Class 2 n = 500, Class 3 n = 285) yielded AUC values greater than chance for Class 1 membership prediction (cross-validated mean AUC = $0.644 \pm0.37$), and Class 3 membership prediction (AUC = $0.543 \pm0.025$), but not for Class 2 (AUC = $0.500 \pm.000$; non-performant model). Using latent class as a predictor of 1-year cessation improved model fit by an average of 17.1% (AUC = $0.767 \pm0.007$ with class assignment as a predictor versus AUC = $0.596 \pm0.003$ without).

Predictors of class membership were largely similar to those reported in the main text with those in Class 1 more likely to be male, have lower anxiety and nicotine dependence, having gone longer without smoking in the past, and having been assigned to active NRT (see sFigure 7).

Latent class assignment remained strongly predictive of smoking cessation at the 1-year follow-up with those in Class 1 as much as five times as likely to achieve cessation as those in Class 2 (cross-validated odds ratio = $0.186 \pm0.179$, ref: Class 1) and six times as likely as those in Class 3 (odds ratio = $0.158 \pm0.16$, ref: Class 1) (see sFigure 8).

This result makes us confident that early smoking behavior (i.e., substantial reduction within the initial weeks of an intervention) is associated with later cessation, and that this relationship is robust to sample size and missing outcomes.


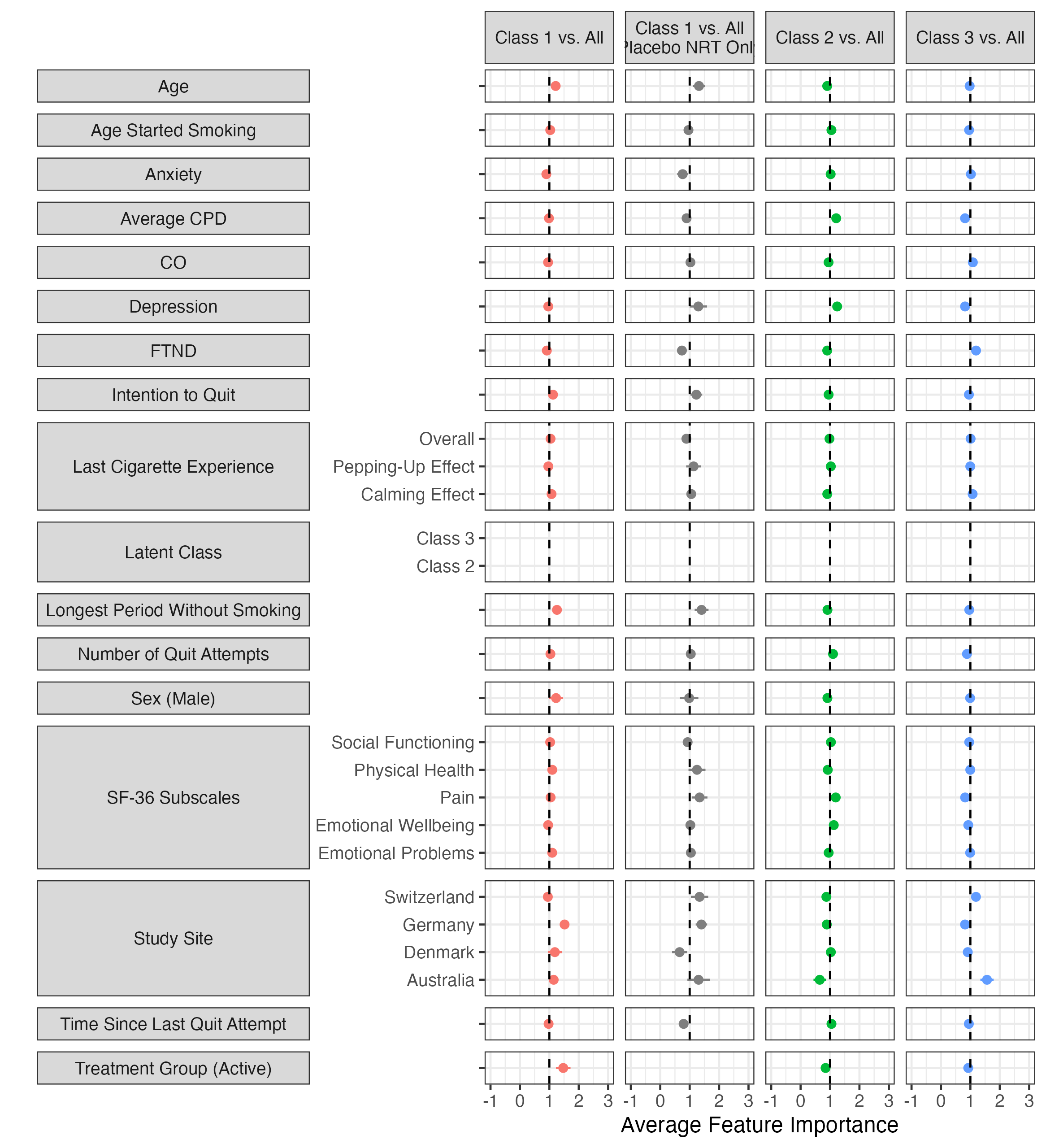


**sFigure 7** Latent class membership prediction odds ratios. Sample limited to participants with recorded 1-year follow-up CO values.


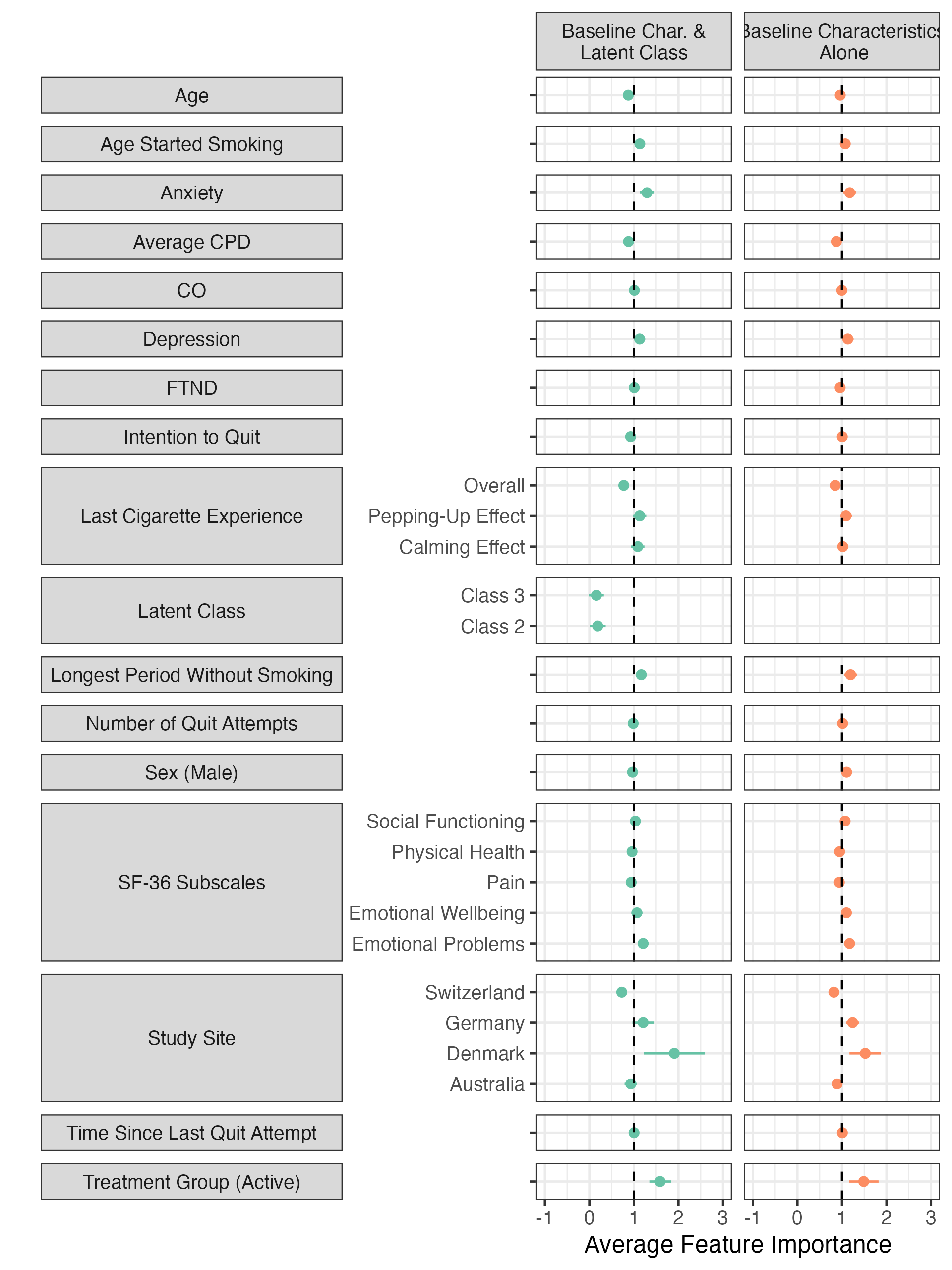


**sFigure 8** 1-year smoking cessation prediction odds ratios. Sample limited to participants with recorded 1-year follow-up CO values.

# Full Analysis with “Never Quit” Baseline Respondents Removed

We observed an inconsistency between trial inclusion criteria (i.e., at least one failed quit attempt) and a small number of participants (n=10) who reported never having quit smoking at their baseline visit. We conducted a sensitivity analysis without these participants and found similar results as the primary analysis for latent smoking trajectories, prediction of class membership (Class 1 mean ROC AUC = $0.645 \pm0.034, p<.001$; Class 2 AUC = $0.512 \pm0.016, p<.001$; Class 3 AUC = $0.562 +/- 0.018, p<.001$, and prediction of 1-year smoking abstinence (mean ROC AUC with latent class as a predictor = $0.793 \pm0.006, p< .001$, without latent class = $0.670 \pm.006, p <.001$.

# Comparison of smoking trajectories under alternative latent class models


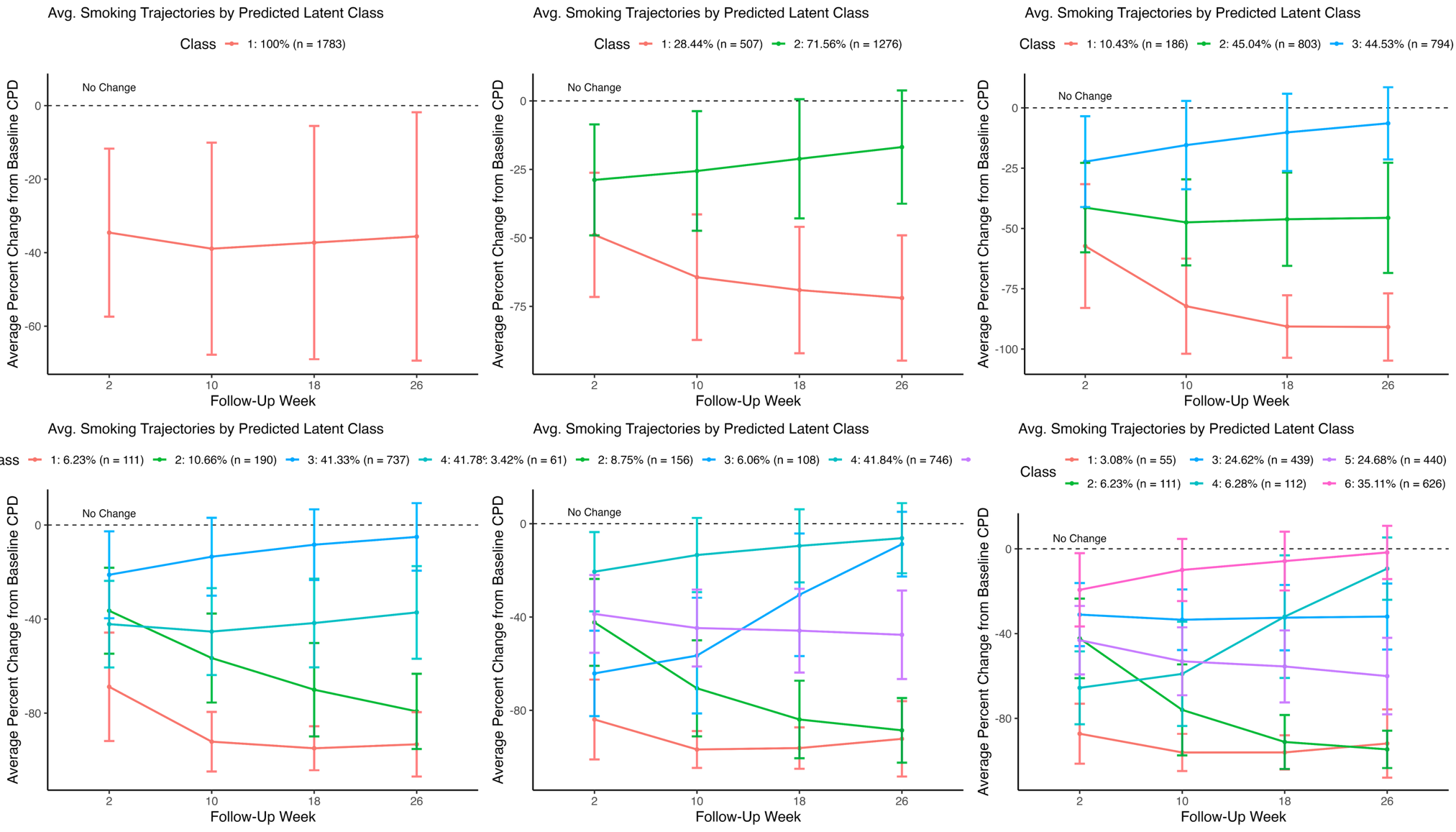


**sFigure 9** Comparison of smoking trajectories across all evaluated latent class models. The three-class model was selected for maintaining model interpretability while also optimizing model fit indices.

# Latent class predictive modeling robustness to alternative data splits


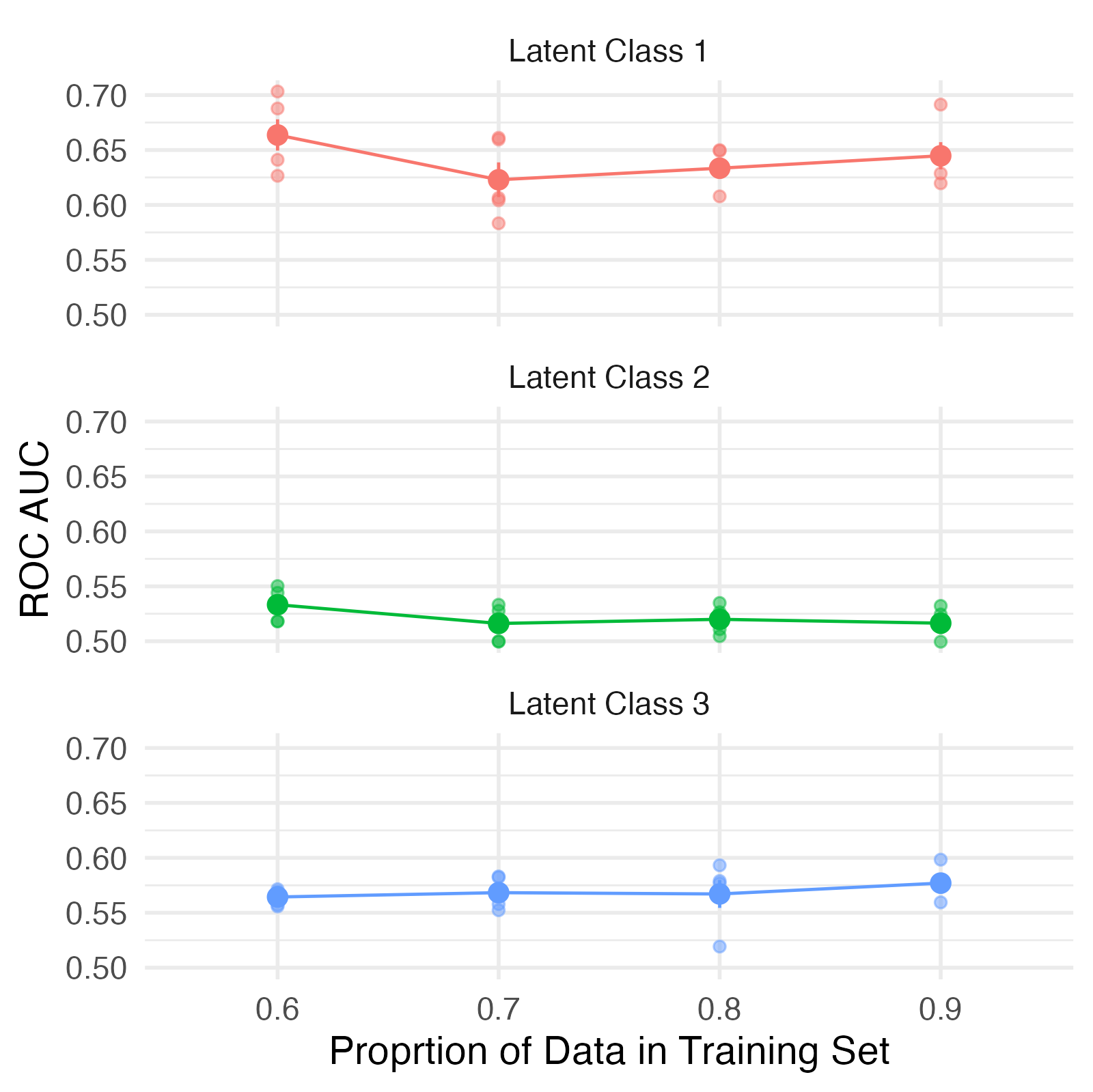


**sFigure 10** Latent class prediction model ROC AUC values across cross-validation folds using progressively larger training sets showing that fit indices are robust to alternative data partitioning. Note: 80% of the data were used for training the models presented in the main text.


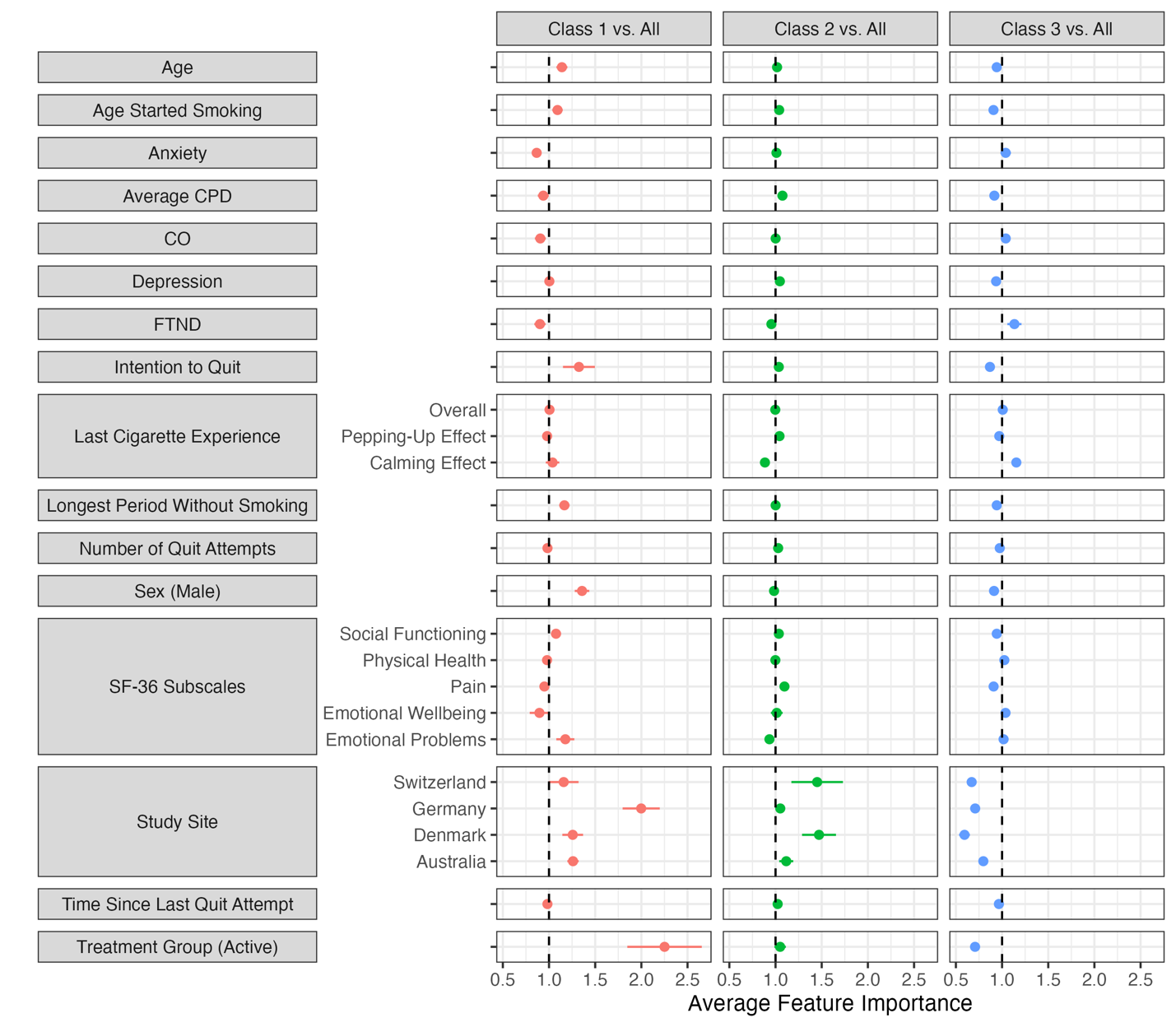


**sFigure 11** Latent class prediction model feature importance values aggregated across four different data partitions (i.e., 60%, 70%, 80%, and 90% used for model training) showing that feature importance is robust to alternative data partitioning.

# References

1. Benowitz NL, Bernert JT, Foulds J, Hecht SS, Jacob P, Jarvis MJ, et al. Biochemical Verification of Tobacco Use and Abstinence: 2019 Update. Nicotine Tob Res. 2020 Jun 12;22(7):1086–97.

2. Hays RD, Morales LS. The RAND-36 measure of health-related quality of life. Ann Med. 2001 Jan;33(5):350–7.
